# Clinical practice guidance for next-generation sequencing in cancer diagnosis and treatment (edition 2.1)

**DOI:** 10.1007/s10147-020-01831-6

**Published:** 2020-11-29

**Authors:** Yoichi Naito, Hiroyuki Aburatani, Toraji Amano, Eishi Baba, Toru Furukawa, Tetsu Hayashida, Eiso Hiyama, Sadakatsu Ikeda, Masashi Kanai, Motohiro Kato, Ichiro Kinoshita, Naomi Kiyota, Takashi Kohno, Shinji Kohsaka, Keigo Komine, Itaru Matsumura, Yuji Miura, Yoshiaki Nakamura, Atsushi Natsume, Kazuto Nishio, Katsutoshi Oda, Naoyuki Oda, Natsuko Okita, Kumiko Oseto, Kuniko Sunami, Hideaki Takahashi, Masayuki Takeda, Shimon Tashiro, Shinichi Toyooka, Hideki Ueno, Shinichi Yachida, Takayuki Yoshino, Katsuya Tsuchihara, Japanese Society of Medical Oncology, Japan Society of Clinical Oncology, Japanese Cancer Association

**Affiliations:** 1 Department of General Internal Medicine/Breast and Medical Oncology/Experimental Therapeutics, National Cancer Center Hospital East, Kashiwa, Japan; 2 Genome Science Division, Research Center for Advanced Science and Technology, University of Tokyo, Tokyo, Japan; 3 Clinical Research and Medical Innovation Center, Hokkaido University Hospital, Sapporo, Japan; 4 Department of Oncology and Social Medicine, Graduate School of Medical Sciences, Kyushu University, Fukuoka, Japan; 5 Department of Investigative Pathology, Tohoku University Graduate School of Medicine, Sendai, Japan; 6 Department of Surgery, Keio University School of Medicine, Tokyo, Japan; 7 Natural Science Center for Basic Research and Development, Hiroshima University, Hiroshima, Japan; 8 Cancer Center, Tokyo Medical and Dental University, Tokyo, Japan; 9 Department of Clinical Oncology, Kyoto University Hospital, Kyoto, Japan; 10 Children’s Cancer Center, National Center for Child Health and Development, Tokyo, Japan; 11 Division of Clinical Cancer Genomics, Hokkaido University Hospital, Sapporo, Japan; 12 Kobe University Hospital Cancer Center, Kobe, Japan; 13 Division of Genome Biology, National Cancer Center Research Institute, Tokyo, Japan; 14 Division of Cellular Signaling, National Cancer Center Research Institute, Tokyo, Japan; 15 Department of Medical Oncology, Tohoku University Hospital, Sendai, Japan; 16 Department of Hematology and Rheumatology, Kindai University Faculty of Medicine, Osakasayama, Japan; 17 Department of Medical Oncology, Toranomon Hospital, Tokyo, Japan; 18 Department of Gastroenterology and Gastrointestinal Oncology, National Cancer Center Hospital East, Kashiwa, Japan; 19 Department of Neurosurgery, Nagoya University, Nagoya, Japan; 20 Department of Genome Biology, Kindai University Faculty of Medicine, Osakasayama, Japan; 21 Department of Obstetrics and Gynecology, Faculty of Medicine, The University of Tokyo, Tokyo, Japan; 22 Section of Information Technology Support, Center for Cancer Genomics and Advanced Therapeutics, National Cancer Center, Tokyo, Japan; 23 Clinical Research Support Office, National Cancer Center Hospital, Tokyo, Japan; 24 Department of Clinical Genomics, The University of Tokyo, Tokyo, Japan; 25 Department of Laboratory Medicine, National Cancer Center Hospital, Tokyo, Japan; 26 Department of Hepatobiliary and Pancreatic Oncology, National Cancer Center Hospital East, Kashiwa, Japan; 27 Medical Oncology, Kindai University Faculty of Medicine, Osakasayama, Japan; 28 Department of Sociology, Graduate School of Arts and Letters, Tohoku University, Sendai, Japan; 29 Department of General Thoracic Surgery and Breast and Endocrine Surgery, Okayama University Graduate School of Medicine, Dentistry, and Pharmaceutical Sciences, Okayama, Japan; 30 Department of Hepatobiliary and Pancreatic Oncology, National Cancer Center Hospital, Tokyo, Japan; 31 Department of Cancer Genome Informatics, Graduate School of Medicine, Osaka University, Osaka, Japan; 32 Division of Translational Informatics, Exploratory Oncology Research and Clinical Trial Center, National Cancer Center, 6-5-1 Kashiwanoha, Kashiwa, Chiba 277-8577 Japan; 33 Present Address: Konica Minolta Precision Medicine Japan, Inc., Tokyo, Japan

**Keywords:** Clinical practice guidance, Cancer genomic profiling test, Next-generation sequencing, Solid cancer

## Abstract

**Background:**

To promote precision oncology in clinical practice, the Japanese Society of Medical Oncology, the Japanese Society of Clinical Oncology, and the Japanese Cancer Association, jointly published “Clinical practice guidance for next-generation sequencing in cancer diagnosis and treatment” in 2017. Since new information on cancer genomic medicine has emerged since the 1st edition of the guidance was released, including reimbursement for NGS-based multiplex gene panel tests in 2019, the guidance revision was made.

**Methods:**

A working group was organized with 33 researchers from cancer genomic medicine designated core hospitals and other academic institutions. For an impartial evaluation of the draft version, eight committee members from each society conducted an external evaluation. Public comments were also made on the draft. The finalized Japanese version was published on the websites of the three societies in March 2020.

**Results:**

The revised edition consists of two parts: an explanation of the cancer genomic profiling test (General Discussion) and clinical questions (CQs) that are of concern in clinical practice. Particularly, patient selection should be based on the expectation that the patient's post-test general condition and organ function will be able to tolerate drug therapy, and the optimal timing of test should be considered in consideration of subsequent treatment plans, not limited to treatment lines.

**Conclusion:**

We expect that the revised version will be used by healthcare professionals and will also need to be continually reviewed in line with future developments in cancer genome medicine.

## About this guidance

With advances in molecular biology, multiple gene alterations related to the acquisition of malignant phenotypes in cancer cells have been identified and are expected to be used to predict the efficacy of drug therapies and to classify, definitively diagnose, and predict the prognosis of cancer. With regard to gene alterations in a diverse range of cancers, as the types of genes being searched for increase, it may be difficult to obtain the specimens needed to search for individual genes, the time involved in testing may increase, or it may be difficult to obtain sufficient information to select the best treatment protocol. The gene panel tests subject to this guidance determine the gene alterations in the cancers of individual patients to provide an opportunity for treatment that is optimal for the characteristics of the cancer. Using a gene panel that can detect multiple gene alterations at one time, the tests analyze gene alterations by means of tools, such as a next-generation sequencer. The gene panel encompasses genes known to be pertinent to drug therapy efficacy, definitive diagnosis, and prognosis prediction and simultaneously provides information on factors, such as gene mutations, deletions, insertions, gene fusion, and gene copy number alterations. This guidance describes the current clinical role of gene panel testing, its foremost objective being to determine treatment strategies for optimal drug therapy by providing information on aspects, such as the feasibility of novel treatments, definitive diagnosis, and prognosis prediction for a variety of cancers, particularly solid tumors for which no effective therapy has been found. Consequently, the guidance gives priority to testing for cancers, such as non-small-cell lung cancer and colorectal cancer, for which companion diagnostics and similar gene-related tests exist. If some of the genes in a gene panel are approved and used as a companion diagnostic, the standard treatment for the companion diagnostic portion is established according to guidelines of the relevant academic society or similar body. Moreover, the scope of gene panel testing may change in the future with developments such as advances in diagnostic and therapeutic technology.

### Background and objectives

According to the National Cancer Center's Cancer Information Service, 995,132 new cases of cancer were diagnosed in 2016 (nationwide cancer registry), and 373,334 people died from cancer in 2017, making it the number 1 cause of death [1]. Consequently, improving the outcomes of cancer therapy is an extremely important issue for the nation. In the field of cancer drug therapy, treatment outcomes and prognoses have improved with the emergence of effective novel therapies. At the same time, the development of biomarkers that identify groups for which efficacy is likely before treatment also have contributed to improving cancer treatment outcomes.

Abnormalities in oncogenes and tumor suppressor genes have been applied to treatment selection as molecularly targeted therapies have been developed, as well as to pathophysiological and etiological diagnosis. The 3rd Basic Plan to Promote Cancer Control Programs (2018) called for building a system that would enable cancer patients to receive genomic medicine anywhere in the country. As a system for implementing genomic medicine, the Expert Meeting for Cancer Genomic Medicine Promotion Consortium Report (2017) published by the Ministry of Health, Labour and Welfare proposed the establishment of cancer genomic medicine designated core hospitals to perform and interpret NGS testing of ensured quality and implement treatment and clinical development. It also recommended that the Center for Cancer Genomics and Advanced Therapeutics (C-CAT) be established to collect and manage clinical genomic information and promote its application in the development of diagnostics and treatment. Based on these recommendations, 11 cancer genomic medicine designated core hospitals, 34 cancer genomic medicine designated hospitals, 122 cancer genomic medicine cooperative hospitals (as of October 2019), and the C-CAT have been established since 2018. In addition, a gene panel testing system received marketing approval in December 2018 and has been covered by national health insurance since June 2019.

To have cancer genomic medicine widely adopted in the clinical setting rapidly and safely, it is useful to have guidance aimed at healthcare professionals that has been edited by a broad range of specialists. In October 2017, three academic societies, the Japanese Society of Medical Oncology, the Japan Society of Clinical Oncology, and the Japan Cancer Association, jointly issued the Guidance for Cancer Treatment Based on Gene Panel Testing Using Next-Generation Sequencers (1st edition). The guidance has influenced areas, such as the establishment of the cancer genomic medicine designated core hospitals in Japan and the development and review of gene panel testing systems. Although the guidance mentions the need for constant revision as developments, such as advances in research and development occur, new information on cancer genomic medicine has emerged since the 1st edition of the guidance was released. This includes information on advanced medical care using various gene panel tests, new gene panel testing systems for which development is progressing in Japan and other countries, and guidelines and recommendations from bodies, such as academic societies and study groups, including the Japan Society of Hematology. Consequently, there is a need to reexamine the information provided in the 1st edition. The decision was therefore made to revise the guidance to contribute to the standardization of gene panel testing. The topics revised concern the necessary considerations for the implementation of gene panel testing (patients to undergo gene panel testing and timing of testing; requirements for medical institutions; evidence level classifications, etc.; test-related information provided to patients; specimen preparation; and expert panel implementation). The revisions are expected to contribute to ensuring that cancer patients throughout the country have the opportunity to receive high-quality genomic medicine care through a network centered on cancer genomic medicine designated core hospitals, etc. They are also expected to be useful in ensuring economically appropriate healthcare by avoiding unnecessary testing.

### Approaches to cancer genomic profiling as a whole

This guidance mainly concerns genomic profiling to detect alterations that occur in solid tumor cells and tissues. Hematologic malignancies are outside the scope of the guidance. This guidance is limited to providing references to separate guidance on hereditary tumors and hematologic malignancies.

Cancer genomic profiling tests currently covered by national health insurance are mainly performed to predict the efficacy of drug therapy. Consequently, they are performed for patients for whom drug therapy is indicated. Depending on the gene panel used in cancer genomic profiling, it may include genes for diagnosis and prognosis prediction that contribute to determining a treatment strategy or genes that have a companion diagnostic function. Consequently, the handling of each type is described. Also described are the timing of the testing, review of the test results by an expert panel, and returning the results.

In referring to the differences in base sequences and structures that occur in genomic DNA, terms, such as "gene alteration", "gene mutation", "genetic abnormality", "variant", and "genomic alteration" are variously used. This guidance mainly uses the term "gene alteration" to refer broadly to changes, such as point mutations, gene amplification, and gene fusion, regardless of whether they are pathologically significant. In cases, such as when a reference that uses a specific term is cited in part, the original term is used.

### Determining the recommendation levels

In preparing this guidance, clinical questions (CQs) were specified, and a systematic review of the literature was performed by manually searching for evidence that provided the basis for the answers to those questions. In searching the literature, the PubMed and Cochrane Library databases were used, and important reports published by a variety of academic societies were also adopted. To determine the recommendation level for each CQ based on the results of the review, panel members voted on the recommendation. Then, based on the results of the voting, the recommendation level for each CQ was established (Table 1). The recommendation level was determined based on factors, such as the strength of the evidence related to each CQ and the anticipated benefits and disadvantages for patients. During the voting, the status of drug approval and national health insurance coverage for the treatment described (including indications for testing and treatment) was not considered but was indicated in a section for remarks when necessary. Recommendation levels were determined as follows based on the voting: (1) strong recommendation (SR) when 70% or more of the votes were for SR; (2) recommendation (R) when the criterion for (1) was not met and the sum of SR + R votes accounted for 70% or more of the votes; (3) expert consensus opinion (ECO) when the criteria for (1) and (2) were not met and the sum of SR + R + ECO votes accounted for 70% or more of the votes; and (4) not recommended (NR) when the criteria for (1)–(3) were not met and NR accounted for 50% or more of the votes. If none of the criteria for (1)–(4) were met, the outcome was "no recommendation".

**Table 1:** Recommendation level and criteria

Recommendation level	Criterion for recommendation level	Description
Strong recommendation (SR)	Strongly recommended because supporting evidence is sufficient and benefits outweigh disadvantages	Strongly recommended
Recommendation (R)	Recommended taking into account the balance of benefits and disadvantages because a certain amount of supporting evidence exists	Recommended
Expert consensus opinion (ECO)	Supporting evidence and information on benefits is insufficient, but a certain level of consensus has been established	Considered
Not recommended (NR)	Not recommended because of no supporting evidence	Not recommended

The recommendations for the CQs include recommendations not based on sufficient evidence currently. The information provided in this guidance and the recommendation levels may change significantly in the future as new evidence accumulates. Although the guidance will be updated as needed, effort should be made to check the latest medical information and use it appropriately when determining whether testing is indicated or using a drug in clinical practice.

## Cancer genomic profiling tests

### Overview of cancer genomic profiling tests

Cancer genomic profiling analyzes all or part of the target genes for changes, such as base substitution/insertion, deletion mutation, gene amplification/deletion, gene fusion, and gene expression, using a next-generation sequencer (NGS). The results are then comprehensively interpreted to select a treatment strategy. In cancer genomic profiling, the genetic changes seen are interpreted as a comprehensive profile, rather than diagnosed using individual biomarkers for which evidence has been established and an approved drug exists, and the results are reflected on treatments other than the standard treatment. This approach makes it significantly different from companion diagnostics, which have played the main role in the conventional genetic testing.

It is mandated that cancer genomic profiling tests be interpreted based on expertise to determine the clinical significance of the results obtained and the treatment selection they indicate. Consequently, treatment strategies must currently be formulated by an expert panel, which are consultative bodies of experts that are convened at cancer genomic medicine designated core hospitals and cancer genomic medicine designated hospitals, and the facilities that use cancer genomic profiling are also limited to cancer genomic medicine designated core hospitals, cancer genomic medicine designated hospitals, and cancer genomic medicine cooperative hospitals.

#### Cancer genomic profiling tests that have been approved or are performed as advanced medical care

Although the number of panel genes is not clearly specified in the definition of cancer genomic profiling tests, the description in the national health insurance listing dated June 1, 2019 indicates as follows: When a comprehensive genomic profile is obtained using solid tumor cells as specimens and a sequencer system that has been approved or certified by the regulatory authority as a medical device used for cancer genomic profiling to detect changes, such as mutations in at least 100 cancer-related genes, the national health insurance point can be calculated only once for each patient by applying the point designated for Category 3 "Tests with extremely complex processing". Two gene panel tests, the OncoGuide™ NCC Oncopanel System (Sysmex Corporation) and the FoundationOne^®^ CDx Cancer Genomic Profile (Chugai Pharmaceutical), are covered by national health insurance as cancer genomic profiling tests. In addition, tests performed under the Advanced Medical Care B Category are the Todai OncoPanel, the applicant medical institution for which is the University of Tokyo Hospital, and the Oncomine™ Target Test System from Osaka University Hospital.

#### Characteristics of each cancer genomic profiling test

The characteristics of the cancer genomic profiling tests performed under regulatory approval or Advanced Medical Care Category B are as follows (Table 2):

1. OncoGuide™ NCC Oncopanel System [2, 3]
  With 114 cancer-related panel genes, this system permits testing for base substitution/insertion, deletion mutation, and amplification in 114 genes and gene fusion in 12 genes, and the tumor mutation burden (TMB). It uses not only tumor tissue-derived DNA but also uses DNA from non-tumor cells (peripheral blood) as a normal control. Consequently, characteristics of the system are that it can also exclude rare genetic polymorphisms and distinguish somatic gene alterations from germline alterations.
2. FoundationOne^®^ CDx Cancer Genomic Profile [4, 5]
  With 324 panel genes, this system enables the measurement of base substitution/insertion, deletion mutation, and amplification in 309 genes and gene fusion in 36 genes, microsatellite instability (MSI), and TMB. A characteristic of the system is that it functions as a companion diagnostic in addition to a cancer genomic profiling test. It enables companion diagnosis for the following therapeutic drugs (as of February 2020): In non-small cell lung cancers, afatinib, erlotinib, gefitinib, and osimertinib for *EGFR* mutations (exon19 del, exon21 L858R; also exon20 T790M for osimertinib); alectinib, crizotinib, and ceritinib for *ALK* gene fusion; and entrectinib for *ROS1* gene fusion; in breast cancer, trastuzumab for *ERBB2* copy number aberrations; in malignant melanoma, dabrafenib + trametinib and vemurafenib for *BRAF* V600E/K mutations; in colorectal cancer, cetuximab and panitumumab for *KRAS/NRAS* wild type; in solid tumors, entrectinib for *NTRK1*/*2*/*3* gene fusion; and in ovarian cancer, olaparib for *BRCA1/2* mutations.
3. Todai OncoPanel [6]
  The Todai OncoPanel analyzes RNA as well as DNA. It consists of 2 panels: a DNA panel with 464 genes and an RNA panel with 463 genes. The DNA panel analyzes somatic mutations in translational exon regions and *TERT* gene promoter regions for 464 genes and can also detect hypermutators and chromosomal copy number aberrations. Major features of the RNA panel are that it not only enables searching for fusion genes and exon skipping but also allows expression level analysis.
4. Oncomine™ Target Test System

**Table 2:** Summary of cancer genomic profiling tests currently being used under national health insurance coverage or Advanced Medical Care Category B

Test name	OncoGuide™ NCC Oncopanel System	FoundationOne^®^ CDx Cancer Genomic Profile	Todai OncoPanel	Oncomine™ Target Test
Specimens tested	Tumor (FFPE) + normal (peripheral blood)	Tumor (FFPE)	Tumor (FFPE) + normal (peripheral blood)	Tumor (FFPE)
Nucleic acid tested	DNA	DNA	DNA, RNA	DNA, RNA
Specimen quantity required	5 μm × 10 specimens (≥ 16 mm^2^ recommended)	If 25 mm^2^: 4–5 μm × 10 specimens (If less, number of specimens adjusted to yield 1 mm^3^)	≥ 15 × 15 mm: 4 specimens ≥ 10 × 10 mm: 6 specimens ≥ 5 × 5 mm: 16 specimens 2–5 mm/4 sides: 20 specimens ≤ 2 mm/4 sides: 40 specimens (thickness, 10 μm)	≥ 25 mm^2^, 10 μm thickness recommended
Specimen eligibility requirement	Tumor cell percentage ≥ 20%	Tumor cell percentage ≥ 30% recommended (minimum ≥ 20%)	Tumor cell percentage ≥ 20% recommended (minimum ≥ 10%)	Tumor cell percentage ≥ 20%
Panel genes	114	324	464 (DNA) 463 (RNA)	46
Items analyzed	Mutation, amplification, fusion	Mutation, amplification, fusion	Mutation, amplification, fusion Expression level [6]	Mutation, amplification, fusion
Companion diagnostic functions	–	Non-small cell lung cancer: *EGFR* exon 19 deletion mutation and exon 21, L858R mutation, *EGFR* exon 20 T790M mutation, *ALK* fusion gene, *ROS1* fused gene	–	–
		Malignant melanoma: *BRAF* V600E and V600K mutation		
		Breast cancer: *ERBB2* copy number aberration (*HER2* gene amplification positive)		
		Colorectal cancer: *KRAS*/*NRAS* wildtype		
		Solid tumors: *NTRK1*/*2*/*3* fusion gene		
TMB	○	○	○	–
MSI	–	○	○	–
Pathological variant of an hereditary oncogene (gene subject to reporting)	○	–	○	–
	(*APC*, *BRCA1*, *BRCA2*, *MLH1*, *MSH2*, *PTEN*, *RB1*, *RET*, *STK11*, *SMAD4*, *TP53*, *TSC1*, *VHL*)		(*TP53*, *BRCA1*, *BRCA2*, *MLH1*, *MSH2*, *MSH6*, *PMS2*, *EPCAM*, *APC*, *MUTYH*, *RB1*, *PTEN*, *MEN1*, *RET*, *STK11*, *VHL*, *SDHB*, *NF1*, *TSC1*, *TSC2*, *CDH1*, *WT1*, *PALB2*)

**Table 3:** Factors to consider with respect to the analytical validity required for genomic analysis using NGS

Category	Recommendation level		
	Required	Recommended	Optional
Sample preparation	Tumor cell percentage DNA* concentration DNA fragment size Library concentration		
Sequence related	Cluster density BQ* score ≥ specified threshold Percentage of valid reads Percentage of reads ≥ specified threshold		
Analysis related	Mapping quality Mean read depth in analysis range Proportion with base depth ≥ specified threshold Percentage of bases with quality value ≥ specified threshold Mean insert size PCR* duplication percentage	Percentage of bases that differ from reference sequence AT/GC* bias	
Mutation related	Depth of mutation loci Mutation quality Allele frequency Strand bias Number of mutations at same locus Number of mutations in specified threshold range		Number of germline mutations Haplotype bias
QC related	Determination of sex in analysis	Estimated contamination percentage	Presence or absence of genotype match Base percentage for mutation loci SNP/indel* ratio Ti/Tv* ratio Homo/hetero ratio •CNV profile

Definitions of abbreviations with an asterisk: *DNA* deoxyribonucleic acid, *BQ* base quality, i.e., value expressing the reliability of bases detected by the sequencer, *PCR* polymerase chain reaction, *AT/CG* adenine (A) and thymine (T) or guanine (G) and cytosine (C), *SNP/indel* refers to a ingle nucleotide polymorphism (SNP) and/or a base sequence insertion or deletion (indel), *Ti/Tv* transversion (Ti), i.e., a mutation between a pyrimidine (C, T/U) and a purine (A, G); transition (Tv), i.e., a mutation between pyrimidines or purines (Modified from Table 4 in Reference [18])

With a total of 46 panel genes, this system uses DNA from tumor specimens to analyze whether mutations are present in 35 genes. In addition, it uses RNA from tumor specimens to analyze fusion genes in 21 genes, particularly *ALK* and *ROS1*. While the systems described in (1)–(3) above use target capture sequencing, this test system uses amplicon sequencing and reports the presence or absence of hot-spot mutations of pathological significance. Because the system is based on multiplex PCR technology, it can perform detection using small samples (10 ng each for DNA and RNA). Moreover, because fusion genes are directly detected by multiplex PCR, an abundance of isoforms, approximately 250 types, can be detected. In Japan, the Oncomine Dx Target Test Multi-CDx System, which is based on the same principle as the Oncomine™ Target Test System, has received regulatory approval. However, it is not a profiling test but rather is covered by national health insurance as a companion diagnostic for non-small cell lung cancer. Consequently, the only results reported are those indicating the presence or absence of *EGFR* gene mutations, *ALK* fusion genes, *ROS1* fusion genes, and *BRAF* V600E mutations. However, upon request from physicians for investigative purposes, the analysis results for all 46 genes may be returned by the testing company as reference data.

### Role of testing

Some gene panel tests not only serve as cancer genomic profiling tests that detect changes, such as mutations of cancer-related genes and provide a comprehensive genomic profile but also have the functions of companion tests for selecting a regimen for treatment with an antineoplastic drug and genomic biomarkers, such as TMB and MSI [7]. When using panels in the clinical setting, the functions provided by a gene panel and the biomarkers that it can and cannot detect are first determined, and the characteristics of each panel are then taken into account.

According to a 2016 report titled Research on Standards Setting for a Cancer Genomic Medicine Provision System (Health and Labour Sciences Special Research Project, Policy Research, Health and Labour Sciences Research Grant; Research Director: Hitoshi Nakagama), the low and high estimates of the number of patients subject to diagnosis of somatic gene alterations are 164,000 and approximately 400,000, respectively. Of these patients, the number predicted to undergo treatment based on the genomic diagnosis is 77,000 and 150,000, respectively [8].

**Estimates of patients subject to cancer genomic medicine (January 31, 2017)**

1. Definition
  Somatic (cancer cell) gene alteration diagnosis.
  Clinical sequencing (i.e., DNA/RNA sequence analysis performed at instruction of attending physician for diagnosis and treatment; results reported to attending physician) or gene expression analysis, and
  Subject to multigene panel or more comprehensive analysis (does not include analysis of single gene or specific hotspot).
  Germline genomic diagnosis.
  Clinical sequencing of multigene panel or more comprehensive analysis.
  Includes single gene analyses.

2. Estimates

**Table:** 

2 Major cancer genomic medicine classifications	Estimate range	Number of patients to undergo genomic analysis	Number of patients subject to treatment or prevention based on genomic diagnosis
Somatic genomic medicine	(1) Low estimate	164,000	77,000 (treatment)
	(2) High estimate	400,000	150,000 (treatment)
(3) Germline genomic medicine		280,000	8000 (prevention)

**Basis of estimates of patients subject to cancer genomic medicine (January 31, 2017)**

1. Basis of estimates
  Low estimates limited to narrow range of indications for somatic gene alteration diagnosis.
  The estimates and totals are only for the following 3 major types of cancer that require early approval and national health insurance coverage.
  A) Colorectal cancer: estimated number requiring "treatment" according to results of "genomic diagnosis" based on the "definitions" above, in reference to cases in GI-SCRUM (Dr. Takayuki Yoshino, National Cancer Center Hospital East [NCCE], January 26, 2017).
  B) Lung cancer: the same as above, in reference to LC-SCRUM (Dr. Koichi Goto, NCCE, January 25, 2017).
  C) Breast cancer: estimated number of patients subject to "genomic diagnosis," in reference to Oncotype DX cases (With Oncotype DX, postoperative adjuvant chemotherapy indications predicted and molecularly targeted therapy/immune therapy, etc., not selected) (Dr. Chikako Shimizu, National Cancer Center Hospital [NCCH]).
  High estimates encompassing broad range of indications.
  In next stage, total number of patients that should be included under the national health insurance system estimated:
  D) All patients desiring chemotherapy for any type of cancer (children and adults) are subject to genomic diagnosis: estimated in reference to TOP-GEAR cases.

2. Estimates
  Low estimates: (patients subject to genomic diagnosis)/(patients for whom prevention and treatment individualized based on the genomic diagnosis information).
  A) Colorectal cancer = 54,320 patients/28,246 patients (According to 2015 NCC data, 135,800 individuals had colorectal cancer. Drug therapy was indicated for 40%, or 54,320, of these patients.)
  B) Lung cancer = 77,000 patients/48,510 patients (Lung cancer occurs in 110,000 patients/year. Of them, non-small cell cancer accounted for 70%, or 77,000 patients, who are the main group subject to genetic screening.)
  C) Breast cancer = 32,290 patients/0 patients (Breast cancer predicted to occur in 90,000 individuals in 2016. In addition, axillary lymph node negative/small number positive, 85%; 67% ER + /HER2 − , 67%; postmenopausal, 63%, etc., for combined total 32,290 patients).
    A) + B) + C) = 164,000 patients/77,000 patients.
  High estimates.
  D) All cancer types = 400,000 patients/150,000 patients (Cancer of any type predicted to occur in 1.01 million people in 2016. Of these, 40% desire chemotherapy. In TOPICS-1, actionable mutation identified in 45%).

#### Views regarding test timing and patients subject to gene panel tests that provide only the function of a cancer genomic profiling test

Approved cancer genomic profiling tests are covered by insurance for patients with solid tumors for which there is no standard treatment for patients with locally advanced or metastatic cancer who have completed standard treatment (includes patients expected to complete the treatment), and who are judged by the attending physician to have a strong likelihood of being suitable for chemotherapy after the test, based on factors, such as their general condition and organ function, according to the chemotherapy guidelines of the relevant academic society. Reimbursement is limited to 1 test per patient in his/her lifetime (as of September 2019). The purpose of cancer genomic profiling tests is to obtain information on genomic alterations related to drug selection, and to investigate a detailed treatment strategy based on the cancer genomic information. However, gene panels that provide functions for examining aspects, such as diagnosis, prognosis prediction, and cancer predisposition are envisaged for the future [9]. When a biopsy can be performed, it is desirable to use the biopsy samples to perform the gene panel test. However, if it is difficult to collect a biopsy sample, a stored specimen obtained at diagnosis, for example, can be used. In this case, whether the stored specimen is suitable for the test should be carefully considered.

The range that the standard treatment for a given disease refers to should be determined by the attending physician for each patient according to the guidelines of the relevant academic society or similar body. The percentage of biomarker-related drugs in the standard treatments is assumed to vary according to the disease [10].

Therefore, the timing of the testing and the suitability of a treatment must be examined by a specialist in the given disease.

#### Views regarding test timing and patients subject to gene panel tests that provide functions of both a companion diagnosis and a cancer genomic profiling test

Some gene panel tests provide the functions of both companion diagnosis and genomic profiling tests. If a gene panel test is approved as a companion diagnosis test for a given tumor type, the gene panel test can be performed at the point where the indication of the drug for the tumor is determined. In this case, the companion diagnosis test results are given priority to the patient when the test results are provided to the attending physician. Under current public health insurance system (as of September 2019), the results of a comprehensive genomic profile obtained in conjunction with an assessment of a specific gene mutation performed to select an antineoplastic drug treatment can be provided to the patient at the time of completion (prospected completion) of standard treatment after being reviewed by an expert panel, along with a written explanation of the treatment strategy, etc. It should be noted that the cost of treatment paid by national health insurance system may differ depending on whether the gene panel test is used as a companion diagnosis or for cancer genomic profiling test. Disclosure of genetic profiling information other than companion diagnosis to patients will be discussed in the different section (CQ12).

#### Matters common to both gene panel tests that provide only the function of a cancer genomic profiling test and those that also provide the function of a companion diagnosis

There have been no reports on the optimal timing for genomic profiling tests in patients with solid tumors, which might be a topic for future investigation. As noted in CQ2, the benefits of cancer genomic profiling tests have not been demonstrated in a randomized controlled study in patients who have completed standard treatment. On the other hand, cancer genomic profiling has been reported to be effective in retrospective cohort and case series studies in patients not limited to those who have completed standard treatment, although the designs and assessment methods of these studies have varied. In view of the fact that treatment options are currently limited after standard treatment is completed, it is considered to provide the patients with treatment opportunities in clinical studies, particularly clinical trials of investigational new drugs, based on the results of NGS testing. Consequently, it is desirable that the patients be in good general condition (e.g., performance status) and have good organ function as a requirement for inclusion in a clinical study, taking into consideration a turnaround time (TAT) of appropriately 1–2 months is needed after tumor tissue is provided for gene panel testing before the results are returned.

Investigations of the significance of repeated cancer genomic profiling when disease progression is seen are also currently ongoing. The *EGFR* T790M mutation in non-small cell lung cancer is a resistance mutation for molecularly targeted drugs and has been shown to be useful in selecting other drugs. Real-time PCR testing of *EGFR* T790M has been approved for patients with non-small cell lung cancer in whom secondary mutation (T790M) of the *EGFR* gene is suspected as a result of recurrence or progression and for whom testing of lung tissue specimens is difficult for medical reasons although the selection of subsequent treatments is required. Genes that should be tested repeatedly during the course of treatment continue to be discovered, such as resistance mutations related to the selection of subsequent treatments and newly acquired driver gene alterations. Based on these findings, the significance of repeated genomic profiling tests should be investigated in the future. On the other hand, repeated gene panel testing using the same specimen is thought to have little meaning and is not recommended as long as there is likelihood that the clinical significance of the repeated testing will be strong for reasons, such as large differences in the genes covered. In the patient suitable for gene panel testing and the testing confirms the presence of an abnormality in a gene for which a companion test exists, a drug can be administered without additional companion testing only in the following cases: an expert panel convened after the gene panel test determines that administration of a drug related to that gene alteration is appropriate based on information sources, such as the package insert, guidelines, or literature; and the attending physician concludes that administration of the drug is appropriate.

No TMB tests, which are used to predict the effectiveness of immune checkpoint inhibitors, or MSI tests, which are used for indication assessment of immune checkpoint inhibitors, have been approved as companion tests included in gene panel tests (as of September 2019). The types of cancer for which a TMB or MSI test and the timing of the test are determined according to the package insert for the gene panel test or the guidelines/guidance issued by the relevant academic society or similar body. For handling of MSI data for NGS, which has not be approved for companion testing, should be referred to the Clinical Practice Guidelines for Tumor-Agnostic Treatments in Adult and Pediatric Patients with Advanced Solid Tumors toward Precision Medicine (Japan Society of Clinical Oncology, Japanese Society of Medical Oncology, ed., with collaboration from the Japanese Society of Pediatric Hematology/Oncology).

### Test implementation system

The 3rd-Term Basic Plan to Promote Cancer Control Programs (2018) called for gradually building a system that would enable cancer patients to receive genomic medicine treatment anywhere in the country. However, it has been pointed out that implementing gene panel tests that have the function of a cancer genomic profiling test poses a variety of challenges that are unlike those faced with companion tests. Consequently, the Ministry of Health, Labour and Welfare held the Expert Meeting for Cancer Genomic Medicine Promotion Consortium in 2017 and, as a result of that meeting, determined that it would be advisable for gene panel tests to be performed at specialized medical institutions (cancer genomic medicine designated core hospitals) that meet the following requirements.

- Have in place a system that permits gene panel testing of sustained quality with respect to aspects, such as the preparation of suitable pathology specimens (outsourcing to organizations, such as testing companies is envisaged as a system for NGS analysis and is permitted)
- Have an expert panel capable of appropriate medical interpretation of gene panel tests
- Have a system for professional genetic counseling
- Have an appropriate system for implementing initiatives in areas, such as clinical studies/trials, including advanced medical care, investigator-initiated clinical trials, and global clinical trials; and have a system to appropriately manage the clinical information obtained from a gene panel test that has a certain level of track record and register such information with the C-CAT.
- Have a track record in diagnosing and treating the patients who subject to gene panel testing.
- Have a system for providing information on subjects, such as genomic medicine to patients and their family members in an easily understood manner.
- Have a system that can freshly cryopreserve biological samples, such as surgical specimens.

These requirements were expressed in specific terms by the Sub-Working Group on Requirements for Designated core hospitals, etc. for Cancer Genomic Medicine (2017). In addition, it was proposed that the system include the establishment of cancer genomic medicine cooperative hospitals, which would provide gene panel testing in collaboration with the cancer genomic medicine designated core hospitals, to optimize patient access, and this was described in a report. Based on the results of this examination, the Ministry of Health, Labour and Welfare established its Guidelines for Establishing Designated core hospitals, etc. for Cancer Genomic Medicine (hereinafter referred to as the "Establishment Guidelines") in December 2017. It designated 11 cancer genomic medicine designated core hospitals in February 2018 and established 156 cancer genomic medicine cooperative hospitals by April 2019. For the 2 gene panel tests with cancer genome profiling test functions that were covered by national health insurance in June 2019, it is required that the test should be performed only by the medical institutions indicated in the Establishment Guidelines.

In April 2019, the Working Group on Requirements for Designated core hospitals, etc. for Cancer Genomic Medicine was convened to reexamine the requirements for medical institutions and the systems involved. The group proposed that, because of the limitations on the processing capabilities of the expert panels imposed by having them only at the 11 cancer genomic medicine designated core hospitals, cancer genomic medicine designated hospitals that conduct expert panels in-house be established. The report was completed in May 2019. Guidelines that reflected the deliberations were issued in July 2019, and in September of that year, cancer genomic medicine designated hospitals at 34 locations were newly designated from among the cancer genomic medicine cooperative hospitals.

### Quality control of specimens provided for gene panel testing

Formalin-fixed paraffin-embedded (FFPE) samples used for routine pathological diagnosis are used in the gene panel test. Although the FFPE sample is a highly versatile tissue resource obtainable in general medical institutions, when it is used for genomic diagnosis, the necessary precautions must be taken in handling the tissue during preparation to obtain high-quality DNA. The Japanese Society of Pathology published its Guidelines on the Handling of Pathological Tissue Samples for Genomic Medicine (March 2018, 1st edition) as guidelines for maintaining the quality of tissue used for genomic medicine. They include methods recommended based on corroborating data, and the following are recommendations based on that and another document [11, 12].

- Collection: Surgically resected tissue is promptly stored under refrigeration at ≤ 4 °C and fixed within 1 h after resection, or within 3 h at the latest. Keeping it at room temperature for more than 30 min after resection should be avoided as much as possible. Tissue collected by biopsy is promptly fixed. Cell samples also can be embedded in formalin-fixed paraffin. In this case, fixation is performed as soon as possible after the necessary pretreatment is performed.
- Fixation: Fixation is performed at room temperature for 6–48 h, using 10% neutral buffered formalin solution at a volume at least tenfold greater than the tissue volume.
- Storage: The FFPE block is stored in a cool, dark place (room temperature permissible), avoiding humidity. Storage as unstained FFPE slices is avoided. When the tissue is provided for genomic diagnosis, slicing is performed.
- Provision for genomic diagnosis: When specimens are provided for genomic diagnosis, an FFPE block with the tumor load required for the analysis is selected by a pathologist in principle, based on observation of HE-stained specimens prepared during pathological diagnosis and information in the pathological diagnosis report. Blocks with bleeding, necrosis, or many non-tumor cells, such as inflammatory cells are avoided to the extent possible. A tumor content percentage of ≥ 30% in a section is desirable, and a minimum of 20% is required. If the tumor content percentage is low, the non-tumor portion is removed by microdissection to increase the content percentage. With tissue sections of 5 × 5 mm in size and a tumor content percentage of 30%, a minimum of ten sections of 4–5 μm in thickness are needed. If the tissue is small, additional sections are required.
- Genomic DNA extraction and analysis: Genomic DNA is typically extracted and analyzed in an analysis laboratory, and the quantity and quality of DNA extracted must meet pre-determined requirements. When the analysis is performed using low-quality DNA, special precautions must be taken in interpreting the data.
- Storage period: for genomic diagnosis, it is preferable to use an FFPE block within 3 years after it is prepared.
- Decalcification: EDTA decalcification is performed, and acid decalcification is avoided.

### Explanation of testing and informed consent

This section describes important points concerning informed consent and the explanations given to patients, including preparing to explain the testing to the patient and addressing a change in a patient's willingness to undergo testing or the withdrawal of informed consent, by reference to the "Draft Informed Consent Procedures" and "Draft Consent Form for Cancer Gene Panel Testing (model document)" prepared by the Informed Consent and Information Utilization Working Group (ICWG) established under the purview of the Liaison Council for Designated core hospitals, etc. for Cancer Genomic Medicine.

#### Preparation for informed consent

- The attending physician will provide the explanation and obtain informed consent. Under the supervision of the attending physician, an explanation assistant*^1^ may assist with in explaining the testing and obtaining informed consent. An appropriate environment should be established and discussions held regarding who will sit with the patient. It is important to determine the patient's level of understanding before the testing.
- Effort should be made to ensure that the patients can independently determine whether to undergo a cancer gene panel test, and they should be given an overall explanation of the testing in advance. In addition, they should be given an opportunity to read and view supplemental explanatory materials and video content if possible.

*1: Explanation assistants should have received training in cancer gene panel testing, particularly in a cancer genomic medicine coordinator workshop.

#### Important points to be explained

**Purpose and significance of testing**

With cancer gene panel testing, a team of specialists comprehensively investigates the characteristics of the cancer cells though genomic analysis and checks for numerous cancer-related gene alterations to determine whether there is a suitable drug or therapy or a clinical study/trial for which the patient may be eligible. The results of the investigation are then conveyed to the patient.

**Patients to be tested**

The patients to be tested will likely vary according to the test used.

The patients to be tested are specified as follows when a cancer gene panel test designated for health insurance coverage on June 1, 2019 is used to obtain a comprehensive cancer genomic profile.

"Of patients with solid tumors for which there is no standard treatment or patients with locally advanced or metastatic solid tumors who have completed standard treatment (includes patients expected to complete the treatment), those who are judged by the attending physician to have a strong likelihood of being suitable for drug therapy after the test according to the guidelines for the clinical practice of the relevant academic society, based on factors, such as their general condition and organ function," in other words, the patients envisaged as undergoing cancer genomic profiling tests are patients with advanced solid tumors for whom it is difficult to propose a drug therapy based on the evidence available following the administration of various standard drug therapies; patients with pediatric, rare, or unknown primary cancers for whom there is little evidence regarding a standard drug therapy; and patients in whom a novel therapy is being investigated, such as in a clinical trial. Little evidence has been established regarding the significance of testing from an early stage in solid tumors subject to standard treatment or pediatric or rare cancers for which there is little evidence for a standard drug therapy. However, the opportunity to use an effective drug from an early stage is not to be overlooked, and the fact that treatment cannot be administered until the test results are obtained is a concern. Consequently, the appropriate timing for testing is a topic for future investigation (CQ6).

**Benefits and limitations of cancer gene panel testing**

Cancer gene panel testing may provide information on drugs, therapies, and clinical studies/trials that are suitable for a patient. However, the likelihood that it will actually reveal an actionable mutation is 27–36% according to reports from other countries and 45–60% according to reports from Japan, although this depends on the type of panel used [3, 13–17]. Moreover, the likelihood that the results will lead to a corresponding treatment is 6–11% according to reports from other countries and 8–13% according reports from Japan. (There have been few cancer studies in children, adolescents, or young adults in Japan, and it is assumed that the likelihood that these groups will receive a treatment based on the results of cancer gene panel testing is even lower than in adults).

The analysis may end in failure depending on the quality and quantity of the specimens used. Consequently, these aspects should be thoroughly explained and the patient's willingness to undergo cancer gene panel testing should be investigated beforehand.

It also should be explained beforehand that even if an appropriate drug is identified, it often cannot be selected as a treatment in cases, such as the following.

- The drug has not received marketing approval in Japan.
- The drug has not received an indication for the type of cancer the patient has.
- The drug has been used only in clinical studies or trials, and the patient does not meet the eligibility criteria.

It should be explained to the patient that if he or she choose not to undergo cancer gene panel testing, he or she will be presented with the best alternatives, including Best Supportive Care (BSC), and will be not placed at a disadvantage with respect to subsequent care.

Because information on hereditary tumors may be obtained, the patient's wishes in cases where secondary findings are obtained, such as findings related to germline variants, will be determined in advance and documented. The possibility that secondary findings could affect not only the patient but also his or her blood relatives should be explained and consented to in advance.

**Testing method**

The specimens used in cancer gene panel testing are tumor tissue or both tumor tissue and normal tissue (blood) from the patient. If a procedure, such as a biopsy can be performed, it is desirable to collect the specimen needed to perform the gene panel test in this way. However, if it is difficult to collect a specimen, a stored specimen obtained at diagnosis, for example, can be used. If a blood specimen is used, new blood is generally collected because fresh blood is required. It should be noted, however, that blood from an allogeneic genome may be contained in blood specimens from patients who have received a transfusion.

Moreover, if the analysis is to be performed overseas, this should be included in the explanation to the patient.

Cancer gene panel testing encompasses not only the analysis of genomic information for tissue, such as tumor tissue but also the process of medical interpretation carried out by specialists (examination by expert panel) based on the analysis results reported by the testing company (see the section on expert panels for the topics examined by the expert panels). The approximate number of days from the start of cancer gene panel testing to the time when the results can finally be explained based on an examination by a team of specialists will be conveyed to the patient in advance.

**Burdens associated with testing**

If a repeat biopsy is performed, the patient may bear the cost and physical burden associated with the biopsy. The physical burden resulting from blood sampling is usually slight.

**Possibility that information on cancer-related heredity (hereditary tumors) will emerge**

With regard to secondary findings (see "Secondary findings or germline findings"), the following will be explained: "the possibility of findings considered beneficial in managing the health of the patient and their relatives and the patient's right not to know the findings after obtaining a thorough understanding of the testing".

It should be explained to the patient that when both tumor tissue and normal tissue are analyzed, secondary findings may emerge, and whether the patient wishes to know the results of that analysis should be determined beforehand.

It should be explained that when only tumor tissue is used in the test, suspected secondary findings may emerge, and whether the patient wishes to know the results of that analysis should be determined. At the same time, it should be explained that if there are suspected secondary findings, a definitive diagnosis will need to be determined separately.

Moreover, it should be explained that secondary findings are genetic information that can be disclosed, and that the absence of secondary findings cannot rule out a hereditary tumor.

Although it is important to determine the patient's wishes regarding the disclosure of the results for secondary findings, the patient may not make a decision until testing is begun. Consequently, it will also be explained in advance that the patient can take their time to decide until the test results are disclosed. If the patient changes their mind regarding the disclosure of secondary findings, they can change their decision from "do not wish to have disclosed" to "wish to have disclosed" or vice versa. In that case, a change-of-consent form will be submitted. During the determination of the patient's wishes and after the results have been disclosed, the patient will be given contact information for genetic counseling.

**Explaining the results of cancer gene panel testing**

Before the test results are disclosed, the patient's wish regarding the disclosure of results related to hereditary tumors will be reconfirmed.

The test results can be conveyed to the patient alone or in the presence of individuals, such as his or her family members. However, the following points should be explained beforehand.

- That the results of the cancer gene panel test can be disclosed to individuals, such as family members of the patient, if the patient consents to disclose the results to them beforehand on the consent form and cannot be told the results directly.
- That the testing will proceed even if permission to share the test results and contact information for family members or similar individuals is not indicated. However, if the sections are left blank, the following points will be explained in advance: that conveying the results to family members, etc., will be difficult even if they want them, that the family members, etc. may be asked about the patient's willingness to have the results disclosed, and that the actual test results will be included in the patient's medical record.
- If family members will be present when the patient is told the results, it is preferable for them to be family members who heard the prior explanation together with the patient.
- To prepare for cases, such as when it is difficult to convey the results directly to the patient, as in the case of a sudden change in the patient's condition or the death of the patient, it should be documented beforehand whether the patient wishes to have their test results explained to someone other than the patient. If the patient does, the name of the individual and their contact information should be documented in advance. With regard to the patient's relationship to the family members, etc., named on the form and the information to be disclosed, the wishes of those family members, etc., and their level of awareness and understanding regarding the disease and patient's condition will be determined and documented in the patient's medical record beforehand.

**Cost of cancer gene panel testing**

Cancer gene panel testing is performed as a health insurance medical service or as advanced medical care. An explanation will be provided in advance regarding when payment will be requested, including the possibility that partial payment may requested if adequate analysis results are not obtained for some reason. In addition, it will be explained that more than one payment request may be issued if the testing provides the functions of both a companion diagnostic and a cancer genomic profiling test.

**Handing of data, etc. used in cancer gene panel testing**

The patient's wishes regarding three items will be determined.

1. Whether the patient consents to providing his or her genomic data and other information to C-CAT in a form that does not directly identify patient
2. Whether the patient consents to providing the data and other information provided to C-CAT to third parties that wish to use it for purposes, such as academic research and pharmaceutical development, after it has undergone a rigorous review
3. Whether the patient consents to having genomic information provided to a company that will perform the analysis or provided by that company to a third party

If the patient consents to (1), information, such as clinical data and genomic information will be entered in a registry at C-CAT, which was established in the National Cancer Center (if the test is performed at a overseas testing company, the genomic information will be sent to that company). In that case, a C-CAT reports will be sent to facilities that hold expert panels, such as the cancer genomic medicine designated core hospitals, to assist with the panels. Consequently, consent for (1) must be obtained at the same time that testing consent is obtained.

It will be explained that even if the patient does not consent to having their information entered in the C-CAT registry, they can still undergo testing, but a C-CAT reports will not be prepared.

With regard to (3), information concerning each company's purpose for using the genomic information and the type of research it is conducting (including sharing with third parties, etc.) will be collected and an explanation provided to the patient as appropriate.

To prepare for the possibility that the patient will change from "consent" to "do not consent" for (1)–(3) above, the following will be explained in advance to avoid misunderstanding.

- That the collection and use of the data and its provision to a third party will be halted after the patient gives notice of their non-consent but that the data will be included in periodic data summaries.
- That the data, including data already provided to a third party, cannot be physically deleted from the database completely.

In addition, that even if the patient gives notice of non-consent to (1), (2), and (3) as whole, the patient can still undergo panel testing and that they can decide on (2) and (3) at a time other than when they decide whether to consent to the testing.

As is indicated in "Protection of personal information" there are cases in which some "genomic data", "genomic information" and "genetic information" handled in the testing may fall under "information requiring consideration". Consequently, organizations, such as the medical institutions performing the tests, C-CAT, and the testing company performing the analyses will exercise adequate caution in handling such data and use networks with sufficient security to exchange data. However, it should be explained at the stage of consent for testing that it cannot be guaranteed that there will be absolutely no disclosure of the data.

#### Withdrawal of consent

The patient can withdraw his or her consent to undergo cancer gene panel testing at any time before the results are disclosed. However, depending on where the testing process stands, the financial charge may not be reduced or canceled.

It should be explained that even if the patient withdraws consent, he or she will not be placed at a disadvantage with respect to subsequent treatment.

It should be explained in advance that the patient will need to submit a change-of-consent form if he or she withdraw his or her consent for any of the following and that the patient can submit this form at any time: the patient's consent to providing his or her information to C-CAT, including genomic and clinical information, to the disclosure of secondary findings, or to the secondary use of his or her data.

#### Informed consent if the patient is a minor

**If the patient is capable of making decisions regarding testing**

As a rule, the consent of a legal representative also will be obtained using a form for adults. The attending physician will decide whether the patient is capable of making such a decision. This does not necessarily need to be determined based on age. Even if the patient is under age 16, the explanation may be provided using a form for adults depending on the patient's ability to comprehend the explanation.

**If the patient is not capable of making decisions regarding testing**

An explanatory document, consent form, and change-of-consent notification for legal representatives can be used. However, the legal representative in this case is generally presumed to be a relative of the patient. If it is not (e.g., a non-blood relative with parental authority or the guardian of a minor), decisions regarding the handling of results related to hereditary tumors are to be made on a patient-by-patient basis.

Even when testing is performed based on the consent of a legal representative of the patient, the "right to know" and "right not to know" in the future, when the patient is capable of making decisions, must be respected. It must be explained to the patient's legal representative beforehand whether the patient will have another opportunity in the future to indicate whether he or she wish to know the results related to hereditary tumors and whether to continue to provide his or her data to C-CAT. However, the purpose of the explanation provided to the legal representative is to ensure that the patient can exercise his or her right to know or not to know in the future; it is not a promise that the healthcare professionals who obtained consent will necessarily create an opportunity to determine the wishes of the patient again.

The patient's wishes should be retained in writing or recorded in the patient's medical record.

### Handling of test results

#### Protection of personal information

The "Act on the Protection of Personal Information" (hereinafter referred to as the "Personal Information Protection Act") was promulgated in May 2003, and full-scale enforcement began in April 2005. The amended Personal Information Protection Act was promulgated in September 2015, and full-scale enforcement began on May 30, 2017.

The Personal Information Protection Act establishes obligations to be observed mainly by private-sector businesses that handle personal information. However, the provisions of "Overall Vision" (Chapter III, Article 1 of the Personal Information Protection Act), "Responsibilities, etc. of the Central and Local Governments" (Chapter II of the Personal Information Protection Act), and "Measures, etc. for the Protection of Personal Information" (Chapter III of the Personal Information Protection Act) also apply to organizations, such as administrative organizations and incorporated administrative agencies as well as to local governments.

The handling of personal information by administrative organizations and incorporated administrative agencies is established by the "Act on the Protection of Personal Information Held by Administrative Organs" (Act No. 58 of May 30, 2003) and the "Act on the Protection of Personal Information Held by Incorporated Administrative Agencies" (Act No. 59 of May 30, 2003), respectively. For the handling of personal information by organizations, such as prefectural governments, governments, boards of education, and public hospitals, the Prefectural or Municipal Ordinances on the Protection of Personal Information established by each local government applies.

On January 22, 2016, a practical promotion task force on health care based on genomic information published a compilation of its views. It referred to an examination of the role of genomic data, etc. in the amended Personal Information Protection Act, based on the fact that genomic data can now be easily obtained as a result of advances in science and technology, such as the emergence of next-generation sequencers, and the fact that distribution is accelerating as a result of developments, such as those in communication technology. The task force defined its terms as follows: "genomic data" are base sequences expressed using character strings, "genomic information" refers to base sequences interpreted to give meaning, and "genetic information" is genomic information that is inherited by offspring. In addition, it mentioned that while genomic data are base sequences expressed as character strings and are of no medical significance as isolated units, genomic information related to matters, such as single gene disorders, predisposition to disease, or drug selection may fall under "information requiring consideration."

Regarding base sequences constituting deoxyribonucleic acid (DNA) taken from a cell, which is designated in Article 1, Item 1-(a) of the Cabinet Order, "genomic data [base sequences constituting deoxyribonucleic acid (DNA) taken from a cell that are expressed as character strings] consisting of heritable information that can be used to authenticate the identity of an individual, such as complete nuclear genome sequence data, complete exome sequence data, whole-genome single-nucleotide polymorphism (SNP) data, sequence data made up of 40 or more mutually exclusive SNPs, repeated sequences of 4 base units (i.e., short tandem repeats [STRs]) at 9 or more loci" are defined as being an individual identification codes by the the Guidelines on the Act on the Protection of Personal Information (General Rules) (November 2016, partially revised in March 2017), and the handling of such data is likely to increase as cancer genomic medicine becomes more widely practiced. Consequently, genomic medicine should be practiced by those with a thorough understanding of the related laws, ordinances, and guidelines.

The "Responses to Personal Data Disclosures, etc. (Notice No. 1 of the Personal Information Protection Commission, 2017)" stipulates the following if by some chance personal information is disclosed. The cases it refers to are as follows: (1) a business that handles personal information discloses, destroys, or damages personal data in its possession (except for specific personal information); (2) A business that handles personal information discloses processing information, etc., in its possession [refers to processing information, etc., as stipulated in Article 20, Item 1 of the Enforcement Rules for the Act on the Protection of Personal Information (Rules of Personal Information Protection Commission No. 3, October 5, 2016), except for specific personal information]; and (3) a risk of (1) or (2) above. If personal information disclosure is detected, the following steps should be taken: (1) in-house reporting of the case and preventing the damage from worsening: immediately report it to the person at the business responsible for reporting and preventing a broadening of the disclosure and take the measures needed to prevent the damage caused by the disclosure, etc. from worsening; (2) determination of relevant facts and investigation of the cause: take the steps needed to determine the relevant facts and investigate the cause of the disclosure, etc.; (3) Determination of the scope of the impact: determine the scope of the disclosure, etc. based on the relevant facts established in (2) above; (4) investigation and implementation of measures to prevent a recurrence: based on the results of (2) above, promptly take the steps needed to investigate and implement measures to prevent a recurrence of disclosure, etc. (5) Notification to those who may have been affected: depending on the nature of the disclosure, etc., promptly notify the individuals affected of the relevant facts or establish a situation where those affected can learn of the relevant facts to prevent secondary harm and the occurrence of similar cases; and (6) announcement of relevant facts and measures to prevent a recurrence: depending on the nature of the disclosure, etc., promptly announce the relevant facts and measures to prevent a recurrence to prevent secondary harm and the occurrence of similar cases.

#### Validity of analysis

Standards and guidelines to be referenced regarding the validity of the analysis will be provided (Table 3).

The following establish detailed standards for cancer gene panel testing in Japan, and testing should be performed in accordance with these standards: "Basic Approaches to Ensuring the Quality and Accuracy of Cancer Gene Panel Testing (Version 2.0)” [18], from the Japanese Promotion Council for Laboratory Testing; and "Views on Quality Assurance Systems for Genetic Testing (Revised in 2018)” [19], from the Japan Registered Clinical Laboratories Association. With regard to the handling of pathology specimens, refer to the section on quality control of specimens provided for gene panel testing.

As is also stipulated in the Medical Care Act, the management of the testing procedures indicated in these references, including personnel training, should be carried out according to established procedures by testing facilities and departments that have received third-party certification, and such management needs to be recorded. In its response to inquiries and interpretations on June 4, 2019, the Ministry of Health, Labour and Welfare stated that "the College of American Pathologists (CAP) standards apply to the third-party certification of accuracy controls for tests that use sequencer systems".

With regard to the data analysis portion, the guidelines of the Association for Molecular Pathology (AMP), etc. have been cited above [20–22]. The AMP guidelines list the following 17 items to examine the validity of the bioinformatics pipeline used to analyze the data, and these must be implemented when performing an analysis in-house [18, 22].

1. Validation will be performed for the bioinformatics pipeline used. This provides a prior understanding of the performance, flaws, and limits of the bioinformatics pipeline. For example, validate should be performed using a reference standard with a known sequence.
2. Validation will be performed under the supervision of an expert in NGS analysis (e.g., the manager of the genetic testing laboratory).
3. Validation will be performed after the bioinformatics pipeline has been designed, developed, brought into conformity, and is well understood.
4. Validation will be performed in the laboratory environment where it will actually be used.
5. Validation will be performed for all of the elements of the bioinformatics pipeline to be used in the analysis, and each element will be reviewed and approved by the responsible supervisor.
6. In the design and implementation of the pipeline, patient personal information will be reliably protected.
7. Validation must conform to the objectives for which the analysis will be utilized (e.g., patients, samples, target genes, variant types).
8. The testing laboratory must guarantee that the design, implementation, and validation of the bioinformatics pipeline are in compliance with the certification standards and regulations of a conforming testing laboratory.
9. The bioinformatics pipeline is part of the analytical method, and its elements and processes must therefore be created and documented according to certification standards and regulations for testing laboratories.
10. Specimen identification must be maintained at each step of the bioinformatics pipeline. That is, misidentification of specimens is not allowed.
11. Parameters for accuracy control and quality assurance must be evaluated through validation and used to ensure satisfactory performance.
12. Sequence data filtering and processing must use validated methods and be accurately documented and recorded.
13. The security of the data in the data files generated by the bioinformatics pipeline must not be disclosed by transfer over networks. That is, problems, such as the disclosure of genomic information or file corruption are not allowed to occur. The safety must be secured and the data integrity must be appropriately guaranteed.
14. In silico validation can be used to validate the bioinformatics pipeline. However, it is not to be used as a substitute for validation using human samples. Basically, proficiency testing will be performed using a FASTQ file for each sample.
15. Bioinformatics pipeline validation will be performed using a representative variant set with high quality that has also been established clinically. In doing so, it is preferable to use reference data generated by a different method. This is because a data set obtained with a similar method will occasionally result in the same errors. The quality standard appropriate for the type of variant should be reported.
16. The testing laboratory must ensure that the documentation of genetic alterations generated by the software used is exactly in accordance with the nomenclature rules of the Human Genome Variation Society (HGVS), which is the international standard for the documentation of genetic alterations, and the accuracy of the annotation information referenced based on the mutation information. In addition, it must conduct appropriate manual reviews and make corrections when necessary, and return correct results.
17. When a pronounced change is made to an element of the bioinformatics pipeline, supplemental validation must always be performed.

The analysis parameters calculated by the analysis pipeline that should be determined to evaluate quality comprise 21 required parameters, three parameters that are not required but are strongly recommended, and eight optional parameters [22].

The evaluation parameters in each category are related to one another, and the part of the overall analysis where a problem has occurred can be investigated according to what type of abnormality is seen in what parameter. An evaluation must be performed for each sample.

Although not all of these data may be presented, before the test is actually conducted, it is necessary to determine what types of quality assessments have been performed. Moreover, the optional parameters also include important parameters, such as the SNP/indel and Ti/Tv ratios, which are related to an examination of sample contamination and an assessment of sample quality. Knowing the type of prior assessment that has been performed provides an understanding of the accuracy of the results obtained. Consequently, the various parameters need to be determined to evaluate the results properly. Useful references include the PMDA's Review Report for each test and the FDA's Summary of Safety and Effectiveness Data.

#### Interpreting the results

**Determining clinical significance**

In their current role in regulatory approval, the knowledge bases referred to in determining clinical significance do not fall in the scope of medical devices that require a regulatory application [23]. Consequently, to provide medical care based on the results of gene panel testing, the type and level of evidence for the interpretation of a detected gene alteration must be noted (for more information, see Item [Evidence levels] in "Evidence levels and types"), and a process whereby clinical significance is determined by comprehensively evaluating various aspects, such as accessibility to drugs and clinical studies, is needed.

**How to determine clinical significance**

The clinical significance of a detected gene alteration should be determined after establishing an appropriate environment, including protocol creation, personnel, and a knowledge base (for more information, see "Knowledge base"). A companion diagnostic for some of the genes in a gene panel has received regulatory approval. If an alteration is detected in such a gene, a decision regarding the use of the relevant therapeutic agent should be made after referring to the Indications and Precautions for the agent.

**Creating a protocol to determine clinical significance**

In determining clinical significance, a protocol that establishes the process for this must be created in advance and shared, and determining clinical significance must be standardized not only within the institutions that perform testing but also between them (for more information, see "Expert panels"). The creation of reports and their scope can be determined according to the guidelines of each testing institution. Consequently, reports may differ even for the same gene alteration. (For example, some guidelines provide for decreasing the amount of information in reports by reporting only gene alterations of obvious clinical significance and not reporting benign variants so that the reports are easy to use for physicians who actually treat patients. On the other hand, guidelines may provide for reporting even gene alterations for which evidence has not yet been established, so that physicians responsible for treating patients can entertain multiple considerations based on the report findings. Because reports can vary greatly depending on the guidelines used, the guidelines should be disclosed to avoid misunderstandings by physicians involved in patient care.)

Testing institutions should establish clear guidelines and disclose the scope of the reporting to the physicians involved in treating patients so that they can understand the differences between the institutions in what they report.

**Individuals who determine clinical significance**

"Knowledge bases," which are essential for determining the clinical significance of the results of gene panel testing, are being developed in both the public and commercial sectors. A knowledge base is a database that organizes information on the types and levels of evidence regarding gene alterations. Individuals who determine the clinical significance of test results must be appropriate specialists who can reach conclusions based on information from multiple resources, such as knowledge bases, databases of on gene alterations in germline and somatic cells, databases of approved drugs and companion diagnostics, clinical study databases, and literature databases. These specialists can determine the clinical significance of a gene alteration by selecting and using appropriate resources based on a thorough understanding of the nature and limitations of the latest new information resources, including knowledge bases.

##### Evidence levels and types

**Evidence levels**

The evidence levels for determining the clinical significance of gene alterations are defined for a different purpose for each knowledge base, and the definition for each must be ascertained when using it. The evidence level for smoothly introducing a patient to a clinical study (use at the development stage) emphasizes gene alterations at the clinical study stage [24, 25]. On the other hand, the definition of the evidence level for clinical use is a standard that emphasizes regulatory approval or the presence or absence of a description in guidelines and prioritizes guiding the patient to a treatment shown to be safe and effective rather than to a clinical study [26].

Even for the same gene and the same alteration of the same gene, the evidence level may differ depending on the evidence type. Information on gene alterations has been utilized in recent years not only to predict treatment efficacy but also for diagnosis and prognosis prediction in the clinical setting. The scientifically valid evidence that has been accumulated and the status of regulatory approval of, for example, drugs or in vitro diagnostics, vary depending on the purpose for which the information is used, such as diagnosis or to predict treatment efficacy or prognosis. Consequently, the evidence level varies depending on the purpose of use even for the same gene and the same alteration of the same gene. Therefore, definitions that can address differences in evidence level according to evidence type are also indicated [25, 27]. In recommendations jointly prepared by 3 academic societies [the American Society of Clinical Oncology (ASCO), the College of American Pathologists (CAP), and the Association of Molecular Pathology (AMP)], treatment response prediction is divided into the categories "sensitivity" and "resistance," and evidence levels are examined to better utilize the information clinically [28]. In addition, the European Society for Medical Oncology (ESMO) has published the ESMO Scale for Clinical Actionability of Molecular Targets (ESCAT). A comparison of the evidence level definitions used in the guidelines from Japan, the United States, and Europe is shown in Table 4.

**Table 4:** Evidence level classifications in guidance issued in Japan, the United States, and Europe

Criterion	Draft revision	Criterion details	Example of measures based on evidence level: draft revision
Response-related evidence level classifications in Japan*^1^			
There is a drug approved in Japan for a specific tumor type	A	There is a drug targeting a specific biomarker approved in Japan for a specific tumor type	If there are drugs approved in Japan, administration of the drug is recommended also based on the results obtained with the companion diagnostic, but in accordance with the various guidelines. Otherwise, the use of evaluation treatments, such as clinical trials, advanced medical care, and the off-label use of a drug listed on the National Health Insurance drug price list, or the use of the system for providing medical expenses combined with treatment outside insurance coverage, such as patient-proposal healthcare services, is recommended, but in accordance with the various guidelines
There is a drug approved by the FDA for a specific tumor type	A	There is a drug targeting a specific biomarker approved by the FDA for a specific tumor type	
A specific tumor type is included in the guidelines	A	The guidelines include the use of a drug targeting a specific biomarker for a specific tumor type	
There have been clinical studies and meta-analyses of high statistical reliability and is a consensus among experts for a specific tumor type	B	Regarding the use of a drug targeting a specific biomarker, there have been supportive data from clinical studies and meta-analyses of high statistical reliability and is a consensus among experts for a specific tumor type	Because there is a scientific basis, the use of evaluation treatments, such as clinical trials, advanced medical care, and the off-label use of a drug listed on the National Health Insurance drug price list, or the use of the system for providing medical expenses combined with treatment outside insurance coverage, such as patient-proposal healthcare services, should be considered
There is a drug approved in Japan or by the FDA for another tumor type	C	There is a drug targeting a specific biomarker approved in Japan or by the FDA for another tumor type	Because there is a scientific basis, the use of evaluation treatments, such as clinical trials, advanced medical care, and the off-label use of a drug listed on the National Health Insurance drug price list, or the use of the system for providing medical expenses combined with treatment outside insurance coverage, such as patient-proposal healthcare services, should be considered
There have been clinical studies and meta-analyses of high statistical reliability and is a consensus among experts for another tumor type	C	Regarding the use of a drug targeting a specific biomarker, there have been supportive data from clinical studies and meta-analyses of high statistical reliability and is a consensus among experts for another tumor type	
Usefulness has been shown in a small clinical study in any tumor type	C	Regarding the use of a drug targeting a specific biomarker, usefulness has been shown in a small clinical study in any tumor type	
Usefulness has been shown in case reports in any tumor type	D	Regarding the use of a drug targeting a specific biomarker, usefulness has been shown in case reports in any tumor type	Although a scientific basis is weak, the use of evaluation treatments, such as clinical trials, advanced medical care, and the off-label use of a drug listed on the National Health Insurance drug price list, or the use of the system for providing medical expenses combined with treatment outside insurance coverage, such as patient-proposal healthcare services, should be considered based on the consensus of the expert panel
Usefulness has been reported in preclinical studies (in vitro or in vivo)	E	Regarding the use of a drug targeting a specific biomarker, usefulness has been shown in preclinical studies (in vitro or in vivo) in any tumor type	Although there is some scientific basis, the use of the drug cannot be aggressively recommended because it has not been administered to humans. However, if the marker is being examined in a clinical trial, enrollment in the trial should be considered based on the consensus of the expert panel. Because the evidence level is expected to increase in the near future, the information is registered with C-CAT
Known to be related to cancerous changes	F	A specific biomarker is known to be related to cancerous changes	Although there is no scientific basis related to treatment selection at this point, if the marker is being examined in a clinical trial, enrollment in the trial is considered based on the consensus of the expert panel. To enhance treatment options by promoting an improvement in the evidence level through the accumulation and utilization of information, the information is registered with C-CAT
Known to be related to drug resistance	R	A specific biomarker is known to be related to drug resistance	Treatment selection is determined based on the consensus of the expert panel

Index summary	Number	Index details
Indices of drug accessibility		
There is a drug approved in Japan for a specific tumor type	1	There is a drug targeting a specific biomarker approved in Japan for a specific tumor type
There have been clinical studies in Japan for a specific tumor type	2	There have been clinical studies in Japan in which a specific biomarker serves as an inclusion criterion for a specific tumor type
There is a drug approved in Japan for another tumor type (off-label use)	3	There is a drug targeting a specific biomarker approved in Japan for another tumor type
There have been clinical studies overseas for a specific tumor type	4	There have been clinical studies overseas in which a specific biomarker serves as an inclusion criterion for a specific tumor type
There is a drug approved by the FDA for any tumor type	5	There is a drug targeting a specific biomarker approved by the FDA for any tumor type
None of the above	6	None of the above apply

*NB* the numbers do not indicate an order of precedence

The status of regulatory approval and national insurance coverage for the drugs and companion diagnostics indicated for a given gene alteration vary according to country. Consequently, evidence levels that incorporate regulatory approval and the recommendations of treatment guidelines in Japan should be used. The evidence levels of knowledge bases currently often adopt approval by the US Food and Drug Administration (FDA) as one standard [29–31]. However, some gene variants approved by the FDA have not received regulatory approval in Japan. Therefore, if an evidence level indicated in a knowledge base is applied unmodified, safety and efficacy in Japanese may not be assured, and the treatment may not be covered by health insurance. Consequently, evidence levels cannot be applied as is to clinical decisions in Japan. It should also be noted that regulatory approval and scientific evidence are not the same. For example, even a drug that has received regulatory approval may no longer be the standard treatment if another drug shown to be more effective in clinical studies has since been approved. With regarding to a treatment's current positioning, the recommendation level should be carefully assessed by consulting the relevant guidelines.

Gene alterations approved as companion diagnostics may also be included in a gene panel test, and this should be kept in mind when selecting a treatment.

**Evidence types**

There are five main types of evidence for the interpretation of a gene alteration.

1. Oncogenic evidence
  This refers to gene alterations that contribute to cancerous changes in cells. In a broad sense, it is a neutral term with respect to whether it refers to somatic mutations or germline variants. However, it is generally used to refer to activated somatic mutations of oncogenes. Somatic mutations are gene alterations that occur specifically in cancer cells, and an important dimension is whether the identified gene alteration is a tumorigenic driver mutation or an incidental passenger mutation.
2. Predisposing evidence
  Germline variants that are related to cancerous changes fall in this category. For example, hereditary breast and ovarian cancer (HBOC) syndrome is a hereditary syndrome associated with a high risk of breast and ovarian cancer that is caused by pathological alterations of the *BRCA1* or *BRCA2* gene in germline cells. It is estimated for account for 5–10% of the 100,000 cases of breast and ovarian cancers that occur annually in Japan [1]. If HBOC is diagnosed, it is recommended that risk-reduction surgery be considered and that the patient's clinical course be observed carefully by means of surveillance to prevent cancer or detect it early.
3. Predictive evidence
  This is of significance for clinical oncology insofar as it pertains to markers of sensitivity and resistance to treatments, such as drug and radiation therapy. It also has pharmacogenomic (PGx) significance, in that it takes into account the possibility of changes, such as the alteration of genes for drug-metabolizing enzymes and drug transporters, which affects pharmacokinetics. If a variant affects drug sensitivity, information indicating whether access to a treatment is possible is important for treatment selection. This includes information, such as whether the variant is a target marker for an approved drug or whether it is a criterion for inclusion in a clinical study.
4. Diagnostic evidence
  This refers to markers related to the diagnosis of patients. Histopathology mainly evaluates the expression and localization of cancer-type-specific marker proteins using immunostaining, in addition to cell morphology and histology, to produce a pathological diagnosis. Gene alterations also aid in diagnosis. For example, extrathyroidal infiltration and distant metastasis are more frequent with the tall cell variant of papillary thyroid carcinoma, a subtype of malignant papillary thyroid carcinoma, than with the well-differentiated type. Consequently, the prognosis is poor and it is highly aggressive. However, *BRAF* V600E positivity is common, making it useful as a diagnostic marker.
5. Prognostic evidence

Markers related to cancer progression, severity and prognosis for survival, etc.

**Indices of drug accessibility**

Even if there is evidence of sensitivity to a drug, the drug will not necessarily have been approved, and its approval status will vary between countries. Moreover, evidence often has not been established for the drug sensitivity of markers specified as eligibility criteria for clinical studies. Consequently, the relationship between a marker and a drug should be evaluated using the index of drug accessibility.

With regard to diagnosis and prognosis, the same criteria were stipulated for Japan and the US based in the US criteria, and they have therefore been omitted here.

*1:Prepared by the Expert Panel Standardization Working Group (EPWG) of the Liaison Council for Designated core hospitals, etc. for Cancer Genomic Medicine.

*2:Standards and Guidelines for the Interpretation and Reporting of Sequence Variants in Cancer: A Joint Recommendations of the Association of Molecular Pathology, American Society of Clinical Oncology, and College of American Pathologists (PMID: 27993330).

*3:ESMO Scale for Clinical Actionability of Molecular Targets (ESCAT), published by the European Society for Medical Oncology (PMID: 30137196).

##### Knowledge bases

Providing treatment based on a gene alteration detected by gene panel testing requires a determination of its clinical significance, which involves establishing an interpretation of the gene alteration and the level of evidence for that interpretation. To do this accurately and efficiently, a knowledge base that accumulates the data to serve as the basis for determining clinical significance and is further developed by the discussions by many experts is useful. A knowledge base is database that not only compiles information indicating whether a known gene alteration is pathogenic but also organizes multiple information resources (e.g., clinical and nonclinical study information, regulatory approval information) related to the clinical significance (response, prognosis, diagnosis) of each gene alteration to present evidence for clinical decision. This concept differs from that of databases, such as ClinVar and COSMIC (the Catalogue Of Somatic Mutations In Cancer), which mainly accumulate information on gene alterations in embryonic and somatic cells. When using a knowledge base, the points below should be kept in mind to choose one that is highly reliable. The main characteristics of publicly available knowledge bases are shown in Table 5.

**Definitions of evidence levels**

The evidence levels for determining the clinical significance of gene alterations are defined differently for each knowledge base, and the definitions for a given knowledge base must be ascertained before using it. Some knowledge bases incorporate the drug approval status in particular into the evidence levels, and it should be noted that such evidence levels may therefore diverge from the scientific evidence. The necessary data should be reviewed to determine the recommendation based on factors such the evidence level, regulatory approval of therapeutic drugs and companion diagnostics indicated for the gene alteration, and insurance coverage.

**Curation**

Curation refers to extracting and organizing data from multiple data resources. In the context of knowledge bases, it refers to extracting data from the literature on matters, such as the regulatory approval status of drugs related to gene alterations, treatment guidance, and clinical and nonclinical (in vivo, in vitro, and in silico) studies and organizing the data according to gene alteration. To choose a knowledge base that suits the objective and provides information of good quality, the following points should be kept in mind.

1. Process
  The curation process at the institution that administers the knowledge base should be determined to confirm that the curation is adequate. To allow this, it is preferable that information on the curation system, such as the method of curation [computed autocuration or manual curation by curator(s)] and the quality of the curator(s) [e.g., academic degrees/qualifications, status of receiving specialized training], be made publicly available. To avoid the risk of incorrectly determining clinical significance based on outdated data, it is desirable that the frequency with which the knowledge base is updated be specified.
2. Reference information
  Curation should encompass the information needed to determine the evidence level (e.g., clinical and nonclinical study data, regulatory approval data). Consequently, the kinds of data resources used to collect the data used to determine the evidence level for each gene alteration should be made explicit.
  Some knowledge bases include references to data from academic conference presentations. Conference presentations are compilations of results obtained up to that point rather than verified analysis data. Consequently, caution is required for gene alterations that reference such information.
  To the extent possible, it is preferable to select a knowledge base that provides metadata on the data sources it references, such as details on gene alteration studies and background information when a variant is detected.
3. Filtering

The curation quality of knowledge base can be considered high if the collected data are periodically filtered. This can involve steps, such as further scrutiny of the data by a specialist or the deletion of outdated data.

If the curation process of a knowledge base is not disclosed publicly, it is desirable to have the accuracy of the data in that knowledge base verified by the individuals who determine clinical significance and expert panel.

New evidence acquired by the individuals who determine clinical significance and expert panels serves as high-quality, valuable information in determining the clinical significance of routine gene panel testing. Collecting such information across testing institutions and compiling it systematically not only increases the quantity and quality of knowledge base data but also contributes to standardizing the method to determine clinical significance by the expert panel at each testing institution. It is therefore preferable for new data to not only be accumulated within expert panels but also shared and compiled across institutions.

NB: In addition to above, knowledge bases, such as the following are also available.

1. The University of Texas MD Anderson Cancer Center: personalized cancer therapy knowledge base for precision oncology. https://pct.mdanderson.org.
2. Vanderbilt-Ingram Cancer Center: my cancer genome. https://www.mycancergenome.org.
3. Institute of Precision Medicine: Welcome to the Precision Medicine Knowledgebase. https://pmkb.weill.cornell.edu.
4. Broad Institute: Welcome to TumorPortal: Genes, cancers, DNA mutations and annotations. http://tumorportal.org.
5. Massachusetts General Hospital: Targeted cancer care. https://targetedcancercare.massgeneral.org.

##### Secondary findings or germline findings

Although cancer gene panel tests are often considered tests of somatic alterations in tumor tissue, they may identify or raise suspicions about germline variants (pathological variants). These are commonly referred as secondary findings or germline findings, although there remains room for future debate regarding the connotations of these terms and what they represent.

Regarding the definition of secondary findings or germline findings, the present guideline employs the definition used in the "Research on Establishing a System for Appropriately Disclosing Genomic Information in the Healthcare Settings in Japan" of the Agency for Medical Research and Development (AMED) (principal investigator: Shinji Kosugi, Kyoto University; hereinafter referred to as the "AMED Kosugi group"), that is, "the finding of a gene mutation in the germline that is definitively pathological" in what is referred to as a cancer gene panel test performed to detect somatic alterations in cancer cells for the purpose of cancer diagnosis, treatment, and prognosis prediction, and follow the views expressed in the "Draft Consent Form for Cancer Gene Panel Testing (model document)" and "Draft Informed Consent Procedure," both prepared by the Informed Consent and Information Utilization WG (ICWG), which was established under the purview of the Liaison Council for Designated core hospitals, etc. for Cancer Genomic Medicine.

**Definition of secondary findings used by the AMED Kosugi group**

For obvious pathological mutations, it is proposed to use the separate terms, i.e., the "primary findings" that are the original objective of the testing and the "secondary findings" in genes that are also subject to analysis. For an overall understanding, see the document at https://www.amed.go.jp/content/000056785.pdf.

It should be noted that the definition differs somewhat from the definition of secondary findings used in the United States Presidential Commission reports and the American College of Medical Genetics (ACMG).

Footnotes:

1. Because "variant" has recently been used instead of "mutation," "variant" is used in the present guidance unless previously defined otherwise.
2. The phrase "in the case of suspected secondary findings that should be disclosed" is used in the guidance to refer to cases where results are obtained that strongly suggest the presence of a pathological variant in the germline even if the panel test uses only tumor tissue.

"Secondary findings" and other related terminology is expected to be changed in the future in conformance with guidelines and guidance issued by bodies, such as academic societies.

Additions and changes may also be made as a consequence of new analysis technology and newly established systems.

Secondary findings can be identified with a fixed probability. Genetic and familial tumors are generally thought to account for approximately 5% of all cancer. However, a recent report of clinical sequencing results showed clinically significant germline mutations in 17.5% of patients with advanced cancer. Of these, 55.5% were not identifiable without testing [32].

**Characteristics of germline genetic information**

The characteristics of germline genetic information are described in the "Guidelines for Genetic Testing and Diagnosis in Medicine (2011)," published by the Japanese Medical Association. Particular attention should be paid to the following characteristics.

- It is unchanged throughout life.
- It is partially shared by blood relatives.
- Genotype and phenotype (note: including future illnesses) of blood relatives can be predicted with a relatively accurate probability.
- Its inappropriate use may have untoward social consequences for the person tested and their blood relatives.

**Disclosing secondary findings**

The genes that are observed for secondary findings vary with the type of panel used for the applicable cancer genomic medicine. Moreover, whether the patient (and his or her family members) wishes to have secondary findings disclosed will be confirmed before the test. Assuming a case in which the patient himself or herself cannot be informed of the results, whether to inform family members of secondary findings will be examined^*1^ It is recommended that whether the patient wishes for the results to be disclosed be reconfirmed when they are disclosed.

Currently, the minimum list of genes suggested for patient disclosure by the AMED Kosugi group includes *BRCA1*, *BRCA2*, *MLH1*, *MSH2*, *MSH6*, *PMS2*, *APC*, *MEN1*, *RET*, *RB1*, and *VHL*, taking into account the following basic considerations:

- There are Japanese guidelines for surveillance of healthy variant carriers.
- Any designated hospital or cooperative hospital for cancer genomic medicine can outsource tests that can analyze only the loci of the specified gene variants to registered clinical laboratories.
- Applicable genes are included in multiple gene panel tests.

However, variations in genes related to hereditary tumors other than these genes also may be found. Consequently, thorough review by an expert panel, etc. is required. This requires a system to practice genetic medicine that, in addition to interpreting the analysis data, can provide the testing, diagnosis, and treatment needed to prevent the diseases that result from secondary findings or detect them early. In addition, this system must provide services, such as those of clinical geneticists and certified genetic counselors, to both patients and the blood relatives.

Moreover, the NCCN guidelines state that genetic testing for *BRCA1* and *BRCA2*, which have a high likelihood of being derived from germline variants, should also be performed in gene panel tests that use only tumor tissue, regardless of factors, such as allele frequency. Hereditary tumors may also be suspected with respect to genes other than *BRCA1* and *BRCA2*, depending on allele frequency, and discussion at an expert panels and collaboration with medical genetics departments must therefore be considered. (Because a hereditary tumor cannot be ruled out based on allele frequency alone with some findings, discussion that encompasses family history and the patient's clinical symptoms may be required.) With panel tests that use only tumor tissue, establishment of a system for testing or outsourcing of testing is required if secondary findings that should be disclosed are suspected and confirmatory testing for a germline pathological variant is needed.

Depending on the circumstances, if the consent form indicates that the patient selects the answer "I wish to be provided with the information" in the item "Provision of cancer-related genetic information (hereditary tumors)" and the answer "The information can be provided to family members or other individuals" in the item "Provision of the results of cancer gene panel tests (included cancer-related genetic information) to family members or other individuals," the results will be conveyed to the individual whose name is entered in the section "Contact information of the person whom you wish to be provided with the results of cancer gene panel tests" of the consent form, and blood relatives need to be provided with genetic counseling.^*2^ The individuals, such as family members, who are to receive the results are limited to those present during the explanation of the cancer gene panel testing.^*3^ The results for secondary findings do not necessarily need to be disclosed at the same time as the primary findings. Rather, the timing of the disclosure of the secondary findings should be based on a comprehensive assessment that takes into account factors, such as the patient's clinical course and family history and the family's circumstances, with both the patient and their family members consulted in this regard. (This is because the surveillance of other organs that is considered necessary as a result of the secondary findings may be of little significance to the patient himself or herself during treatment for cancer.)

Footnotes:

*1: Because information on hereditary tumors may be obtained, the patient's wishes in cases where secondary findings are obtained, such as findings related to germline variants, will be determined in advance and documented. The possibility that secondary findings could affect not only the patient but also his or her blood relatives should be explained and consented to in advance.

*2: The following points pertain to genetic counseling for blood relatives:

1. To ensure that patients and their blood relatives for whom secondary findings are obtained are linked to periodic surveillance, etc., and that information is shared among a more extensive group of blood relatives, genetic counseling is to be provided to such individuals continuously at appropriate times.
2. A system that can implement genetic tests to determine whether blood relatives have the same mutation is to be established.

*3: The test results can be conveyed to the patient alone or in the presence of individuals, such as their family members. However, the following points should be explained beforehand.

- The results of the cancer gene panel test will be disclosed to individuals, such as family members of the patient, if the patient consents to this beforehand on the consent form and cannot be told the results directly.
- Testing will proceed even if permission to share the test results and contact information for family members or similar individuals is not indicated and those sections are left blank.
- That sharing the results with family members will be difficult even if they want them if that section is left blank, that the patient may be asked about his or her willingness to have the results disclosed to family members, etc., and that the test results will be included in the patient's medical record.
- If family members will be present when the patient is told the results, it is preferable for them to be family members who heard the prior explanation together with the patient.

**Important points regarding secondary findings in cancer genomic medicine**

Information on secondary findings obtained in cancer gene panel testing is limited (limitations of the test). Even if at this point any variant type that could lead to a disease is not observed in a candidate gene that is the cause of a hereditary tumor, the correlation between this hereditary tumor or cancer and a genetic cause cannot be ruled out. Moreover, cancer gene panel testing cannot substitute for a genetic test that is mainly intended to analyze germline pathological variants.

If secondary findings are obtained, it is essential that an in-hospital system be established that permits collaboration with the medical genetics departments of each institution. When whole-genome sequencing is performed instead of panel tests in the future, pathological variants in genes other than those related to cancer, such as genes involved in cardiovascular disease, may also be found, and how to handle such cases should be taken into account.

Footnotes:

The sections on secondary findings in this guidance follow the recommendations of the AMED Kosugi group. Medical institutions with systems that provide cancer genomic medicine require an understanding of the following passage from those recommendations.

"Treatment has begun based on the results of genetic diagnosis of hereditary breast and ovarian cancer syndrome and on the results of microsatellite instability testing, which may be used in screening for Lynch syndrome. Germline mutations in these conditions are close to the primary findings for treatment and are more important than other secondary findings. Thus, it should be kept in mind that the definition of a hereditary tumor as a secondary finding in cancer gene panel testing is becoming ambiguous."

#### Expert panels

While a lot of genomic information may be obtained by cancer gene panel testing, it cannot be utilized in treatment unless it can be accurately interpreted. Consequently, a process called an "expert panel" in which specialists from multiple disciplines meet and clinically interpret genomic information is essential. The expert panel examines the obtained genome data, using information, such as the results of C-CAT reports and reports of gene panel tests for reference, while taking into account the patient's background. Based on the discussions by the expert panel, the attending physician is responsible for explaining the findings to the patient and then finally determining a treatment strategy.

##### Panel members

Under the Ministry of Health, Labour and Welfare's "Guidelines for Establishing Designated core hospitals, etc. for Cancer Genomic Medicine" (partially revised on July 19, 2019), the requirements for the membership of an expert panel are as follows.

1. It must include multiple full-time physicians in different fields of organ who have specialized knowledge and skills related to cancer drug therapy.
2. It must include at least one physician with specialized knowledge and skills related to medical genetics.
3. It must include at least one individual with specialized genetic counseling skills related to medical genetics.
4. It must include multiple physicians with specialized knowledge and skills related to pathology.
5. It must include at least one specialist with a thorough knowledge of molecular genetics or cancer genomic medicine. Such a specialist would preferably have authored a peer-reviewed English-language article (limited to first author or corresponding author) on cancer genomic medicine or cancer genome research within the 3 years prior to the time of application.
6. If sequencing is to be performed internally at the institution, the expert panel must include at least one specialist with the thorough knowledge of bioinformatics needed for genetic analysis performed using next-generation sequencers. Such a specialist would preferably have authored a peer-reviewed English-language article (including coauthor) on cancer genomic medicine or cancer genome research within the 3 years prior to the time of application.
7. If an institution examines pediatric cancer patients internally, the expert panel must include at least one physician with specialized knowledge of pediatric cancer who has previously participated in an expert panel.
8. The attending physician of the patient to be examined at the expert panel or a substitute for the attending physician.

In addition to representatives from the above fields, it is desirable to have active participation in the expert panel by medical personnel, such as physicians, pharmacists, nurses, and clinical laboratory technicians, who are involved in cancer genomic medicine.

##### Conferences

Conferences are held by cancer genomic medicine designated core hospital s (hereinafter referred to as "designated core hospitals") or cancer genomic medicine designated hospitals (hereinafter referred to as "designated hospitals"). The attending physician of the patient to be examined or a substitute for that physician must participate in the conference from the cancer genomic medicine cooperative hospital (hereinafter referred to as "cooperative hospital"). Some cooperative hospitals are geographically distant from a designated core hospital. In that case, secure Web conferencing may be used at the discretion of each designated core hospital, taking into account the frequency of conferences, discussed below, and the substantial burden that meeting face-to-face would place on cooperative hospital healthcare professionals in terms of time and economics. If Web conferencing, cloud storage, or online information-sharing tools are used, systems that comply with the following three guidelines from the three ministries indicated must be adopted: "Guidelines for Security Management of Medical Information Systems, 5th edition" (Ministry of Health, Labour and Welfare); "Guidelines for Security Management in Information Service Providers Contracted to Manage Medical Information" (Ministry of Economy, Trade and Industry); and "Guidelines for Security Management in Handling of Medical Information by Cloud Service Providers" (Ministry of Internal Affairs and Communications).

Conferences should be held approximately once a week so that patients are not placed at a disadvantage by a delay in receiving their test because of having to wait for a conference. However, because the number of cancer gene panel tests performed is predicted to increase rapidly in the future, it is expected that making decisions by circulating information using technology, such as cloud storage and online information-sharing tools, will be permitted for patients in whom cancer gene panel tests do not detect a mutation that could lead to treatment (evidence level of C or higher in the separate table "Evidence levels and types"). Moreover, if patients must wait for conferences due to an increase in the number of cases examined, it will be important to prioritize patients by taking into account their general condition and treatment status.

In doing so, each institution will be responsible for preparing and retaining lists of conference participants and managing personal medical information. Conference participants are prohibited from disclosing the personal information of patients to third parties.

In view of the organizational and personnel burden imposed by collaboration between hospitals, it will be desirable in the future to have a system that works to both foster personnel who meet the requirements of institutions, such as cancer genomic medicine designated core hospitals, and ensure that such personnel are stably available to conclude matters within their own institution.

##### Matters to be considered by expert panels

The expert panel will consider the following points, using the C-CAT reports and the reports of cancer gene panel test results as reference data.

*Overall test*

A) Quality of specimens and data (particularly when sequencing performed internally at the institution)

*Each gene alteration*

B) Determination of the biological significance of the gene alteration (e.g., whether it contributes to the acquisition of a specific phenotype, such as potential for malignant transformation)
C) Whether there is a candidate therapeutic drug for the gene alteration
D) The panel will examine possibilities, such as whether there are any recommended gene alterations and specific candidate drugs or clinical studies addressing such gene alterations that should be given precedence, taking into account evidence levels and the patient's background (e.g., age, performance status, comorbidities).
E) Interpretation of evidence related to diagnosis and prognosis
F) If there are secondary findings (or the suspicion of such), a determination of their significance and a response to the findings will be examined as described in "Secondary findings or germline findings".

The biological significance of detected gene alterations is addressed in a report by Richards et al. for germline alterations and in a report by Li et al. for somatic alterations [28, 33]. Both reflect the joint consensus of the US three academic societies: the College of American Pathologists (CAP), Association for Molecular Pathology (AMP), and American Society of Clinical Oncology (ASCO). The external databases for investigating biological significance that are referenced by these reports are public databases, as indicated in the relevant FDA guidance [34]. The databases used and their versions must be documented in the analysis results.

The specialists who constitute the expert panel and their roles (A-F above) are summarized in Table 6.

**Table 5:** Main knowledge bases

Knowledge base	Cancer Driver Log (CanDL)	Cancer genome interpreter	CIViC	OncoKB
Administrator	Ohio State University	Barcelona Biomedical Genomics Lab	Washington University School of Medicine	Memorial Sloan Kettering Cancer Center (MSKCC)
Year opened to public	2015	2015	2015	2016
Purpose	To provide researchers, molecular pathologists, and bioinformaticians with a simple approach that allows rapid annotation based on driver mutations for which there is direct evidence regarding functional characteristics in literature	To rationalize and automate the entire cancer genome interpretation process	Targets a broad audience, including researchers, clinicians, and patients Purpose is to provides a consistent way to more simply interpret a variety of published data Moreover, to enable precision medicine by disseminating knowledge regarding the clinical significance of genetic variants in cancer and provide an educational forum for vigorous discussion	To create a clinical support tool that is accessible not only to specialists but to all physicians at any medical institutions and that eliminates unneeded data from the variety of data and converts the data to a standardized form that is readily interpretable. In doing so, to support clinicians in interpreting gene alterations in cancer and selecting the optimal treatment for the individual patient
Curation methods	Specialists in the field Autocuration + manual curation Partial autocuration to reduce processing time for large datasets	Specialists in the field Manual curation Date are accumulated, curated, and interpreted based on a standard operating procedure investigated under the H2020 MedBioinformatics project	Crowd-sourcing + specialists in the field Manual curation Creating an account as a curator, editor, or administrator enables users to participate in submitting, adding, and amending data	Specialists in the field Manual curation
Curation quality assurance	Data published after autocuration and subsequent manual review by at least 2 specialists	Clinical and research community provide feedback for editing current entries and make new entries	Editors accepts or rejects additions and revisions made by curators (published after collaboration by at least 2 individuals)	An individual hired as a curator curates the data under the supervision of MSK clinicians and clinical researchers who specialize in the diseases or genetics of various fields
Login required/not required to view data	Not required	Not required	Not required	Not required
Public API	Not applicable	Not applicable	Applicable	Not applicable
Reference information	Scientific literature	Information from publicly available databases (Cancer Gene Census, DoCM, ClinVar, OncoKB, Gene Drug Knowledge Database, IARC TP53 Database)	Information from publicly available databases (PubMed, PubChem, Disease Ontology, Sequence Ontology, EntrezDB)	Scientific literature FDA, NCCN, and ASCO guidelines Clinical Trials.gov Academic conference presentations
Number of entries (as of April 25, 2018)	Genes: 62 Variants:	Validated oncogenic alterations: 5601 Biomarkers of drug response: 1631	Genes: 346 Variants: 1844	Genes: 477 Variants: 3855
Coverage Subjects	Somatic cells	Somatic cells	All variants	Somatic cells
Definitions of evidence levels	Tier 1: Variants that match Food and Drug Administration (FDA) approval or treatment recommendation of National Comprehensive Cancer Network (NCCN) Tier 2: Variants that match the treatment based on evidence from clinical studies, case reports, or exceptional responders Tier 3: Variants that predict response/resistance to treatment based on evidence from nonclinical study data (in vivo or in vitro models) Tier 4: Variant presumed to be an oncogenic driver based on functional activation	[Cancer Biomarkers database] Clinical Practice Indication approved by FDA or recommendation from an international organization, such as the NCCN, etc Late Clinical Trials Example: Phase III–IV Early Clinical Trials Example: Phase I–II Clinical case reports Pre-clinical data	Level A: Validated association Consensus established for treatment of humans Level B: Clinical evidence Suggested by clinical studies or other early patient data Level C: Case study Individual case reports published in the scientific literature Level D: Preclinical evidence Suggested by in vivo or in vitro models Level E: Inferential association Evidence types Response predictions Prognosis predictions Diagnosis Predisposition NB: Indicates combination of evidence level and evidence type	Level 1: Biomarker recognized by FDA that predicts response to therapeutic drug approved by FDA for a specific type of tumor Level 2A: Standard-of-care biomarker that predicts response to therapeutic drug approved by FDA for a specific type of tumor Level 2B: Standard-of-care biomarker that predicts response to therapeutic drug approved by FDA for a different type of tumor Level 3A: Although persuasive clinical evidence supports that a biomarker predicts the response to a therapeutic drug for a specific type of tumor, neither the biomarker nor the therapeutic drug are standard-of-care Level 3B: Although persuasive clinical evidence supports that a biomarker predicts response to a therapeutic drug for a different type of tumor, neither the biomarker nor the therapeutic drug is standard-of-care Level 4: Although persuasive biological evidence supports that a biomarker predicts response to a therapeutic drug for a specific type of tumor, neither the biomarker nor the therapeutic drug is standard-of-care Level R1: Standard-of-care biomarker that predicts resistance to therapeutic drug approved by FDA for a specific type of tumor Level R2: Although persuasive clinical evidence supports that a biomarker predicts resistance to a therapeutic drug, neither the biomarker nor the therapeutic drug is standard-of-care Level R3: Although persuasive biological evidence supports that a biomarker predicts resistance to a therapeutic drug, neither the biomarker nor the therapeutic drug is standard-of-care
Advantages	Can determine whether it is an FDA-approved drug or standard treatment based on the evidence Curation manually reviewed by experts	Linked to other databases and knowledge bases Shows therapeutic drugs that address a specific gene variant References that provide the evidence are shown	Evidence levels distinguishing between diagnosis, response prediction, and prognosis prediction are shown For response prediction, evidence levels distinguishing between response and resistance are shown Shows therapeutic drugs to treat a specific gene variant References that provide the evidence are shown	Evidence that distinguishes between response and resistance is shown Can determine whether it is an FDA-approved drug or standard treatment based on the evidence level References that provide the evidence are shown Curation supervised by MSK clinicians and researchers
Disadvantages	Does not reflect regulatory approval, guidelines, etc., in Japan Small numbers of genes and variants Evidence level definitions do not distinguish between diagnosis, response prediction (response/resistance), and prognosis prediction Cannot identify the corresponding therapeutic drug without checking the references	Does not reflect regulatory approval, guidelines, etc., in Japan Evidence level definitions do not distinguish between diagnosis, response prediction (response/resistance), and prognosis prediction How the quality of curators or curation is ensured is not known because the qualifications of curators or the status of feedback from the community is unclear	Does not reflect regulatory approval, guidelines, etc., in Japan Because anyone can submit data, it is unclear how the qualities of curation and annotations are ensured, and bias in the data is a possibility	Does not reflect regulatory approval, guidelines, etc., in Japan No classifications of evidence levels for diagnosis
URL	https://candl.osu.edu/	https://www.cancergenomeinterpreter.org/biomarkers	https://civicdb.org/home	http://oncokb.org/#/
References	Damodaran et al. J Mol Diagn. 2015 Sep; 17(5): 554–9	Tamborero et al. Genome Med. 2018 Mar 28;10(1):25	Griffith et al. Nat Genet. 2017 Jan 31; 49(2): 170–4	Chakravarty et al. JCO Precis Oncol. 2017 Jul; 2017

##### Handing of personal information and data

As is indicated in "Protection of personal information", personal information and data will be handled according to the relevant laws and regulations. The information system will be constructed and operated and personal information and data handled in accordance with laws and regulations, such as the Act on the Protection of Personal Information and the three guidelines of three ministries (Ministry of Health, Labour and Welfare; Ministry of Economy, Trade and Industry; and Ministry of Internal Affairs and Communications).

First, the following items are required in response to the Ministry of Health, Labour and Welfare's "Guidelines for Security Management of Medical Information Systems."

1. Providing information to C-CAT and receiving C-CAT reports
2. Recording information provision to C-CAT

Some personal information and source genomic data will be provided to C-CAT for patients who consent to such information provision. The institutions that provide the information must therefore store records in electronic medical records, etc. In doing so, the individual who entered the information and the time it was entered must be recorded.

B) Halting information disclosure at the patient's request

When the patient requests that their information no longer be provided to C-CAT and withdraws their consent to allow C-CAT to provide their information to third parties, C-CAT must be notified and appropriate changes made to the C-CAT system. Such changes have been formulated.

C) Encrypting the provided information

Designated core hospitals have implemented data encryption (patient or clinical information, source genomic data) on their systems. Cooperative hospitals have encrypted source genomic data and will not transfer patient or clinical information by entering the data directly into the C-CAT system. Designated hospitals plan to implement information system changes based on those of designated core hospitals.

When source genomic data are transferred from a genome sequencing company (registered clinical laboratory), the data will be encrypted using systems equivalent to those of designated core hospitals.

D) Encrypting the communication channel

The responsibility for the pathway used to communicate with C-CAT when sending it data lies with the institution. When the institution is receiving data, the responsibility for the pathway lies with C-CAT.

Designated core hospitals have implemented advanced encryption using an L2 VPN and the IPsec VPN protocol. Cooperative hospitals have implemented encryption of the communication channel using the IPsec VPN protocol and built a network with increased security in the inter-network connection between the internet and the C-CAT closed network.

The pathway used to transfer source genomic data from a genome sequencing company (registered clinical laboratory) is an encrypted network equivalent to those used by designated core hospitals.

(2) Expert panels

Expert panels must be provided with information, such as case summaries and test results reports. To conduct expert panels efficiently, a remote conferencing system, such as a TV or Web conferencing system, is also used.

Use of a cloud service via the internet is also envisaged for this purpose. Consequently, compliance is required with the "Guidelines for Security Management in Information Service Providers Contracted to Manage Medical Information" (Ministry of Economy, Trade and Industry) and "Guidelines for Security Management in Handling of Medical Information by Cloud Service Providers" (Ministry of Internal Affairs and Communications), in addition to the Ministry of Health, Labour and Welfare guidelines.

A) Information-sharing tools

Although designated core hospitals have often considered using the cloud-based information-sharing tools of a certain company that were easy to use and had security features, it was found that some of the features used overseas servers and therefore did not conform to the Ministry of Health, Labour and Welfare guidelines.

On the other hand, C-CAT has built a cloud-based system for some of the data it receives from cooperative hospitals and therefore prepared an information-sharing tool tailored to this system that is used by expert panels and conforms to the 3 guidelines of 3 ministries.

B) Remote conferencing systems

Most expert panels assume to use Web conferencing systems, and therefore they must conform to the three guidelines of three ministries. Some designated core hospitals have prepared on-premises (self-owned) Web conferencing systems instead of using a cloud service. Some cloud-based Web conferencing services have been found to use overseas servers only for video recording and to use servers in Japan for their other features. Consequently, there have been examples of using such services for expert panels without using the video recording feature.

#### Reports

##### Report preparation

**Reports by testing institutions (including C-CAT reports)**

The gene panel test reports prepared by the testing institutions and C-CAT reports ("reports from testing institutions" below) should include the following information.

- Genes, sequence range^a)^, and types of anomalies^b)^ covered
- Disease name, organ from which specimen collected, date specimen collected, tumor cell percentage^c)^
- Test date
- Quality of specimen DNA, sequence, etc.
- Details regarding detected gene alterations,^d)^ specimen in which detected^e)^
- Determination of biological significance of detected gene alteration^f)^
- Specific candidate drug(s) for gene alteration and evidence level
- Candidate drug indication status and availability rank based on clinical trial information^g)^
- Presence or absence of secondary findings and determination of their significance
- Scope of reports
- Databases used to determine significance^h)^ and dates accessed
- A point to consider: the determination of clinical significance is not complete but may change in the future.
- Entire coding region or a specific region of the gene
- Whether fusion, amplification, TMB, MSI, etc., are included. For amplification, its definition.
- Indicate that a portion of the specimen was dissected if it was.
- Types of anomalies, including variant allele frequency (VAF) (for gene alterations)
- Distinguish between somatic cell and germline origin
- Pathological mutations, etc.
- Treatment accessibility
- Polymorphism databases, knowledge bases compiling evidence for candidate drugs, etc.

Note: For hematopoietic neoplasms, drug selection and the indication of stem cell transplantation are determined based on the evidence for gene alterations related to diagnosis and prognosis in some cases. Consequently, the evidence for gene alterations related to diagnosis and prognosis from the Japanese Society of Hematology's "Guidelines for Genomic Testing for Tumors of Hematopoietic and Lymphoid Tissues" should also be included.

**Reports by expert panels**

Interpreting the results of a gene panel test requires a high level of expertise because the platform varies between tests. The expert panel conducts a review based on the latest information and the status of each patient, using the testing institution reports and C-CAT reports for reference. It then either compiles the results of the review in a written report or documents them in medical records, etc.

The following information should be provided in the expert panel's report.

- Whether there is a recommended treatment and a description of any such treatment
- Treatment options other than the recommended treatment
- Whether there are germline mutations for which an explanation to the patient is recommended and descriptions of any such variants
- Information in material, such as the testing institution report, that is judged to require revision or elaboration
- Sources used as basis

In addition, it is advisable to include the following points.

- That although the expert panel bases its review on the treatment history of the patient, it considers treatments other than the standard treatment, and that the decision to administer the standard treatment is the responsibility of the attending physician.
- That the conclusions of the expert panel are based on the scientific knowledge and clinical study information currently available and may change as new information is obtained in the future.

The attending physician will consider a treatment strategy based on the results of the expert panel together with information, such as the testing institution report. The report or medical record that documents the discussion of the expert panel is intended to serve as a report for the attending physician, and disclosure of this information to the patient will be conducted according to the procedures specified by each medical institution.

##### Returning reports

The report from the testing institution and the C-CAT reports will be provided to the medical institution for use as reference data by the expert panel. The results of the gene panel test will be explained to the patient by their attending physician or another physician substitute for the attending physician, based on the expert panel's discussion. If a treatment strategy, such as participation in a clinical study or trial can be proposed, an explanation of the study treatment also will be provided. The explanation will be documented in the medical record. The expert panel's report (if one is prepared) will be disclosed to the patient in a manner determined by the individual medical institution.

For the procedure for disclosing secondary findings, points to consider in disclosing secondary findings specified in the "Recommendations for Information Transfer Processes in Genomic Medicine, Part 1: Focus on Cancer Gene Panel Testing (revised version)" will be used as a reference. The recommendations were prepared under the AMED Genomic Drug Discovery Infrastructure Promotion Research Project A-(2) titled "Research on Establishing a System for Appropriately Disclosing Genomic Information in the Healthcare Settings in Japan" (principal investigator: Shinji Kosugi, Kyoto University). The patient's wishes regarding disclosure will be carefully determined, and if secondary findings that should be disclosed are suspected in a panel test that examines only tumor tissue, a test to confirm the secondary findings should be performed after explaining the test to the patient again and obtaining their consent. If it is established that there are secondary findings that should be disclosed, consideration should be given so that they will be disclosed at a location where privacy is ensured, under the condition where adequate genetic counseling can be provided by appropriate staff, including personnel, such as a clinical geneticist and certified genetic counselor. The information disclosed for secondary findings will be what the expert panel determines should be disclosed based on the minimum list indicated in the above-referenced recommendations (see "Secondary findings or germline findings").

Even if gene panel testing is performed, there is a strong likelihood that no treatment option can be proposed, or even if one is proposed, accessing the treatment may be very difficult. As was noted earlier, in view of the fact that secondary findings need to be addressed, it should be ensured that there is adequate time to explain the test results, and an environment should be prepared that allows privacy. In addition, effort should be made to have family members present if possible. There is a strong likelihood that the results to be conveyed will differ from what the patient expects. Consequently, supportive communication techniques are also needed, such as SPIKES and SHARE, which are techniques to use in conveying bad news [35, 36].

##### Report handling

The report prepared by the testing institution, the C-CAT reports, and the expert panel report will be retained as medical records. The C-CAT reports will be prepared as reference data for the expert panel and will not be provided to the patient. How the expert panel report is used will be determined by the individual medical institution.

The expert panel report is considered a medical record and will therefore be subject to the disclosure of medical records. The disclosure of medical records will be handled according to the "Guidelines for Providing Medical Records (Notification No. 0912001 of the Health Policy Bureau, Ministry of Health, Labour and Welfare)."

### Genetic counseling

**What is genetic counseling?**

Genetic counseling is a process that assists those with a hereditary disease and their family members and associates to understand the disease's medical and psychological effects and effects on family members and to adapt to these effects. This process involves the following.

- Interpreting the family history and medical history to assess the likelihood that a disease will occur or recur.
- Providing education on heredity, testing, management, prevention, resources, and research.
- It includes aspects, such as informed choice regarding the risks and circumstances (autonomous choice based on sufficient information) and counseling to encourage adaptation.

A healthcare system needs to be created that can implement these requirements when a germline variant is observed or suspected (including secondary findings) in cancer genomic medicine.

Moreover, before cancer genomic medicine is implemented, a healthcare system must be established that makes genetic counseling available for patients and family members who wish to have a detailed understanding of genetic effects and germline variants.

It is important that genetic counseling not only assess the risk of hereditary disease related to the observed variant but also lead to an appropriate medical understanding and surveillance subsequently, including dealing with healthy variant carriers in the family. To do this, a system must be established for follow-up that includes risk-reduction surgery and surveillance.

**System to provide genetic counseling for cancer genomic medicine**

As is indicated in the AMED Kosugi group's "Recommendations for Information Transfer Processes in Genomic Medicine," if the presence of a pathological germline variant is established or suspected:

- It should be disclosed to patient at a location where privacy is ensured, under the condition where adequate counseling can be provided by appropriate staff, including a clinical geneticist or certified genetic counselor.
- There should be cooperation between departments and specialists in and outside the institution that are concerned with the disease involved in the secondary findings.
- The results for secondary findings do not necessarily need to be disclosed at the same time as the primary findings. Rather, the timing of the disclosure of the secondary findings should be based on a comprehensive assessment that takes into account factors, such as the patient's clinical course and family history and the family's circumstances. (This is because the surveillance of other organs that is considered necessary as a result of the secondary findings may be of little significance to the patient himself or herself during treatment for cancer.)
- To ensure that patients and their blood relatives for whom secondary findings are obtained are linked to periodic surveillance, etc., and that information is shared among a more extensive group of blood relatives, genetic counseling is to be provided to such individuals continuously at appropriate times.
- A system that can implement genetic tests to determine whether blood relatives have the same mutation is to be established.

A system for cooperation among these genetic medical care systems and departments needs to be established.

The patient is the first choice to inform blood relatives of secondary findings useful for health management.^*1^ However, depending on the condition of the patient, healthcare professionals may convey this information.^*2^ Whether the family members are notified by the department attending physician or the medical genetics department will be determined for each individual patient, taking into account the relationship between the healthcare professionals and the patient and their family members and the need to explain the patient's condition.

A system that provides the following supports, as well as continuous counseling for the patient, their family members, and blood relatives, is also required.

1. To ensure that patients and their blood relatives for whom secondary findings are obtained are linked to periodic surveillance, etc., and that information is shared among a more extensive group of blood relatives, genetic counseling is to be provided to such individuals continuously at appropriate times.
2. A system that can implement genetic tests to determine whether blood relatives have the same variant is to be established.
3. Continuous support must be provided for the patient and their family members. This should include introducing the patient and their family to a consultation center and a psychological support structure (e.g., clinical psychologist, palliative care team) established at the medical institution.

Footnotes:

*1: "Secondary findings useful for health management" are findings that may reduce the risk of cancer by means, such as risk-reduction surgery or surveillance, or may be linked to early detection and treatment. However, whether they are actually used in health management depends on the healthcare system of the particular country. Twenty-four diseases (currently 27 diseases, 59 genes) are indicated in the recommendations of the American College of Medical Genetics (ACMG) as diseases that should be disclosed to the patient because a treatment or method of prevention is available. However, actionability varies depending on a variety of circumstances, and the ACMG's 59 genes cannot yet be determined to be actionable in Japan. Consequently, the current reference for such information is the "Grade 1 Minimum List for Patient Disclosure of Secondary Findings from Cancer Gene Panel Tests," released by the AMED Kosugi group. At present, the Sakurai subgroup of the AMED Kosugi group is continuously examining the response to secondary findings. In addition, a permanent working group was established to examine the handling of secondary findings in the working group established under the purview of the Liaison Council for Designated core hospitals, etc. for Cancer Genomic Medicine. It is considered that actionable gene alterations will continuously expand through the examination of efforts aimed at actionability and its implementation in Japan.

*2: The test results can be conveyed to the patient alone or in the presence of their family members. However, the following points should be explained beforehand.

- The results of the cancer gene panel test will be disclosed to individuals, such as family members of the patient, if the patient consents to this beforehand on the consent form and cannot be told the results directly.
- Testing will proceed even if permission to share the test results and contact information for family members or similar individuals is not indicated and those sections are left blank.
- That sharing the results with family members will be difficult even if they want them if that section is left blank, that the patient may be asked about his or her willingness to have the results disclosed to family members, etc., and that the test results will be included in the patient's medical record.
- If family members will be present when the patient is told the results, it is preferable for them to be family members who heard the prior explanation together with the patient.

**System for providing genetic counseling to patients who are children, adolescents, or young adults**

Hereditary tumors account for a higher proportion of cancers in children, adolescents, and young adults than in adults. For pediatric patients and adolescent and young adult patients under 20 years of age, a parent is more likely to receive genetic counseling than the patient.

An explanation should be given in a comprehensible manner so that the patient also understands the disease to the extent allowed by their age, and age also will be taken into account when disclosing the type of cancer.

If a secondary finding is observed in a cancer patient who is a child, adolescent or young adult, the likelihood that the patient's blood relatives are unaffected variants with a pathological mutant increases. Consequently, a system of genetic counseling staffed by personnel well versed in cancer in children, adolescents, and young adults needs to be established for blood relatives diagnosed as healthy variant carriers as a result of secondary findings.

It is envisaged that cancer gene panel testing in cancer patients who are children, adolescents, or young adults will be performed with the consent of an adult blood relative acting as the patient's legal representative. In that case, whether secondary findings are provided to the patient will also be determined by the legal representative. However, information must be provided to the patient on their "right to know" and "right not to know" when the patient reaches an age where a patient who is a child, adolescent, or young adult can fully understand matters, such as the nature of the test and the disclosure of secondary findings.^*3^ Accordingly, genetic counseling must also be provided at the stage where patient wishes to know the results.

**Providing genetic counseling and healthcare**

Based on the germline variants seen in cancer genomic medicine, it is anticipated that some blood relatives of patients will wish to undergo genetic counseling and genetic testing.

Consequently, a system that can provide such counseling and testing regardless of whether cancer is present will be essential.

If it proves difficult to establish an in-hospital system for follow-up of unaffected variants, including measures, such as risk-reduction surgery and surveillance, it will be ensured that appropriate medical management is implemented through steps, such as providing information on facilities that can provide these services.

Because blood relatives may live far from an institution that performs cancer gene panel testing, a system for cooperation is required that includes providing information on facilities equipped to provided genetic healthcare where patients can be consulted.

There are limits to the germline variants that can be observed with cancer gene panel testing. Consequently, even if there are no secondary findings with a cancer gene panel test, a germline variant may be present. Therefore, if the patient wishes to have secondary findings disclosed and the attending physician suspects a hereditary tumor based on clinical symptoms but there were no secondary findings in the cancer gene panel test, the patient should be informed that the possibility of a hereditary tumor cannot be ruled out. The patient also needs to be told that genetic counseling is available if the patient desires it. Thus, a healthcare system that can provide genetic counseling is also required in this case.

The germline variant seen may be a variant of unknown significance (VUS) rather than a pathological variant or a variant suspected of being pathological. In that case, if a hereditary tumor is suspected clinically or based on family history, the option of referral for genetic counseling will be considered as part of a thorough examination by the expert panel.

Footnotes:

*3: Determining the wishes of patients who are children, adolescents, or young adults.

**If the patient is capable of making decisions regarding testing**

As a rule, the consent of a legal representative also will be obtained using a form for adults. The attending physician will decide whether the patient is capable of making such a decision. This does not necessarily need to be determined based on age. Even if the patient is under age 16, the explanation may be provided using a form for adults depending on the patient's ability to comprehend the explanation.

**If the patient is not capable of making decisions regarding testing**

An explanatory document, consent form, and change-of-consent notification for legal representatives can be used. However, the legal representative in this case is generally presumed to be a relative of the patient. If it is not (e.g., a non-blood relative with parental authority or the guardian of a minor), decisions regarding the handling of results related to hereditary tumors should be made on a patient-by-patient basis.

Even when testing is performed based on the consent of a legal representative, the patient's "right to know" and "right not to know" must be respected in the future when the patient is capable of making decisions. At that point, the patient will be asked whether he or she wish to know results related to hereditary tumors and whether to continue providing their data to databanks, such as C-CAT. This also must be explained to the patient's legal representative in advance.

However, the purpose of the explanation provided to the legal representative is to ensure that the patient can exercise their right to know or not to know in the future; it is not a promise that the healthcare professionals who obtained consent will necessarily create an opportunity to determine the wishes of the patient again.

The patient's wishes should be retained in writing or recorded in the patient's medical record.

## Reference information

### Personnel development

The practice of cancer genomic medicine requires the involvement of many healthcare professionals, and developing such personnel is an urgent challenge. Cancer genomic medicine was included in the "Enrichment of Cancer Care" item of the "Sectoral Policies" section of the "3rd Basic Plan to Promote Cancer Control Programs," which was decided by the Cabinet in March 2018, and one of the areas to be addressed was "Promotion of Developing the Personnel Needed in Cancer Genomic Medicine." The Ministry of Education, Culture, Sports, Science and Technology has promoted establishing educational programs, mainly for physicians but also for professionals, such as nurses and pharmacists, to address the development of cancer genomic medicine personnel in what are referred to as professional courses in the postgraduate curricula of universities. The Ministry of Health, Labour and Welfare, on the other hand, is promoting the "Cancer Genomic Medicine Professional Training Project," as lecture-based training program for nurses, pharmacists, and clinical laboratory technologists. In the Practical Research for Innovative Cancer Control initiative, which is supported by an AMED grant, personnel development and e-learning programs have been established by the Nishio and Yoshino groups, and it is anticipated that the personnel trained in these programs will be deployed to cooperative hospitals. However, despite these educational programs, there are still shortages of the clinical laboratory technicians needed to perform genomic medicine-related tests, the nurses needed to deal with patients, the pharmacists needed to dispense the molecularly targeted drugs administered based on the genomic data, and the bioinformatics specialists who are essential for preparing genetic diagnostic reports. Establishing and arranging qualification systems certified by academic societies is particularly indispensable to setting career paths for the personnel who implement genomic medicine. Consideration must also be given to development courses for certified genetic counselors and bioinformatics specialists, who do not have qualifications as healthcare professionals, and to specifying their positions when they work at medical institutions. The establishment of a close-knit network of educational institutions, relevant academic societies, and medical institutions is therefore considered necessary.

The current counseling system for genomic medicine has been established in a manner that diverts clinical geneticists and certified genetic counselors for this purpose. Rather than being intended strictly for cancer genomic medicine, these qualifications were originally aimed at establishing a healthcare system for providing accurate information regarding the significance of the results of genetic and other tests and approaches to coping with disease predictability. The annual number of new cancer patients reached 1.01 million in 2016, and as the application of gene panel testing grows due to its coverage by healthcare insurance, the number of tests performed is expected to increase dramatically. In response to the expected increase in the number of patients, quantitative and qualitative improvements in the counseling system will be essential. A particularly urgent challenge will be developing the personnel involved in genomic medicine. However, there is little prospect of a rapid increase in the number of certified genetic counselors because graduation from a master's degree program is required to qualify. Consequently, in 2017, the Ministry of Health, Labour and Welfare began a training program (cancer genomic medicine coordinator workshops) that envisages healthcare professionals, such as nurses, pharmacists, and clinical laboratory technologists employed at institutions, such as cancer genomic medicine designated core hospitals, being involved in work, such as the following.

- Before gene panel testing: explaining panel tests to patients, explaining the possibility that there will be secondary findings.
- After gene panel testing: coordinating trials, etc., and setting up genetic counseling on secondary findings.

### New technology

The liquid biopsy is anticipated as a minimally invasive or noninvasive method of genomic testing. The term liquid biopsy refers to an analysis of tumor-derived specimens (cells or nucleic acids) obtained from the blood or other body fluids. Of these specimens, circulating tumor DNA (ctDNA) has already been partially used for clinical application.

Because specimens for minimally invasive ctDNA analysis can easily be collected during the course of treatment, a new gene alteration acquired as a mechanism of resistance to a molecularly targeted drug can be detected. *EGFR* T790M mutation caused by EGFR inhibitors (non-small-cell lung cancer), *RAS* mutations and *MET* amplification by anti-EGFR-antibody drugs (colorectal cancer), and *ESR1* mutation by aromatase inhibitors (breast cancer) are representative acquired alterations, which has increasingly been elucidated. The Cobas^®^ *EGFR* mutation detection kit, which detects *EGFR* mutations of non-small-cell lung cancer, and the OncoBEAM RAS CRC Kit, which detects *RAS* mutations of colorectal cancer, have been approved in Japan as companion diagnostics.

In addition, a more accurate understanding of gene alterations immediately before treatment is anticipated with ctDNA analysis. In the randomized phase III BELLE-2 study, which examined the efficacy of the PI3K inhibitor buparlisib in patients with hormone receptor-positive, HER2 negative breast cancer, *PIK3CA* mutation in tumor tissue and ctDNA was investigated [37]. In 64 of the patients in that study, the *PIK3CA* mutation was not detected in tissue specimens at diagnosis, and the patients were positive for the *PIK3CA* mutation only in ctDNA during study participation. A trend toward an increase in progression-free survival (PFS) was seen in these 64 patients with treatment, including treatment with buparlisib. In the randomized, phase III SOLAR-1 study, which examined the efficacy of another PI3K inhibitor alpelisib, the drug was shown to be effective [38]. Based on this result, the therascreen^®^ PI PIK3CA RGQ PCR Kit was approved by the FDA in May 2019.

ctDNA analyses are broadly divided into PCR-based assays that analyze a limited number of genes, such as the kits that have received regulatory approval mentioned above, and assays based on next-generation sequencing (NGS), which are used for genomic profiling tests. NGS-based assays are divided into those that use amplicon-based sequencing and those that use capture-based sequencing. Capture-based sequencing is more comprehensive than amplicon-based sequencing, but its limits of detection are an order of magnitude worse (single-base mutations: 0.25–0.5%). Amplicon-based sequencing, which has an improved limits of detection, measures a small number of genes [39]. The limits of detection of both methods are improved using molecular barcoding and bioinformatics techniques to eliminate errors [40, 41]. High concordance rates have been reported in recent studies, including sensitivity and specificity of 90% or greater in a comparison with an analysis using tumor tissue specimens in non-small-cell lung cancer [41].

Although the American Society of Clinical Oncology and College of American Pathologists, in summarizing the previous literature as of 2018, stated that there were insufficient evidences to recommend the use of NGS-based ctDNA assays in clinical practice, subsequently, the number of reports that showed its clinical efficacy has increased [42, 43].

Assays of microsatellite instability (MSI) and the tumor mutation burden (TMB), which are useful for treatment selection, as well as gene mutations and amplification, that can detect mutations at high concordance rates with assays of tumor tissue specimens have been developed [44, 45]. A report describing the first 100 patients in the TARGET study indicated that actionable mutations were detected in 41 patients and that 11 patients received a matched therapy [46].

In a prospective cohort study comparing the detection of resistant genetic alterations with cell-free DNA (cfDNA) and tissue biopsy in 42 patients with gastrointestinal cancers that had become post-treatment resistant, resistance genetic alterations were detected in 32 of the 42 patients (76%) by cfDNA analysis [47]. In 23 patients, in whom both of tissue specimens and cfDNA collected after drug resistance emerged were examined, resistance genetic alterations were detected in 11 patients (48%) using tissue specimens and 20 patients (87%) using cfDNA, suggesting the usefulness of cfDNA analysis for detecting such resistance alterations.

As with gene panel tests that use tumor tissue, NGS-based ctDNA assays are anticipated to be useful for selecting the subsequent therapy based on acquired resistance-related genomic aberrations, in addition to determining a treatment strategy based on cancer genomic profiling tests. Particularly when considering a treatment change due to treatment resistance, testing with a minimally invasive ctDNA assay offers a major advantage in view of the physical burden that a repeat biopsy places on the patient. In addition, an acquired resistance-related genomic aberration may emerge for each treatment, as in the case of the osimertinib resistance mechanism in *EGFR* T790M mutation-positive non-small-cell lung cancer, suggesting that it is meaningful to perform multiple tests to assess the resistance mechanism.

However, the following points should be kept in mind with regard to ctDNA analysis.

1. There are no uniform standards for matters, such as blood specimen collection; blood collection tubes; specimen handling, storage, or transport; DNA extraction or purification; or the evaluation of analytical or clinical validity [42, 43].
2. If the amount of ctDNA in the blood is insufficient, it may not be detectable. As an example, factors that have been reported to reduce the ctDNA detection rate in colorectal cancer include a low tumor load, previous primary tumor resection, lung metastasis, peritoneal dissemination, mucinous adenocarcinoma, and receiving drug therapy [48, 49]. In addition, limits of detection may be deteriorated depending on the localized site of the primary or metastatic tumors and the type of gene alteration (e.g., detection of fusion genes).
3. Gene alterations associated with clonal hematopoiesis of indeterminate potential (CHIP) are known to occur with age. Although most DNAs with these CHIP-related gene alterations have a low allele frequency, there is no established method of rigorously distinguishing it from ctDNA [50].

In addition to the characteristics mentioned above, cancer genomic profiling tests using ctDNA offer advantages, such as reducing the time involved in specimen preparation. On the other hand, the present limitations also should be noted, such as deteriorated limits of detection due to the cancer type and therapeutic modification. A testing method should be selected after appropriately assessing the usefulness of testing using tissue and liquid biopsy for the individual patient.

#### Whole-genome sequencing

Although international, comprehensive and exploratory genomic research has been conducted by international consortia, such as the International Cancer Genome Consortium (ICGC), investigating genome changes in cancer is a future priority for Japan. The "Basic Policy on Economic and Fiscal Management and Reform 2019," announced by the Cabinet Office on June 21, 2019, states the following: "In order to put in place a mechanism to accumulate genome information in Japan, and to promote research for cancer drug discovery and personalized cancer treatment with use of the whole-genome analysis, aiming to overcome cancer, and for early diagnosis of intractable diseases by the whole-genome analysis, in reference to the UK that implemented the examination of the whole-genome of 100,000 people and aims to examine that of one million people, the government will formulate a specific implementation plan, including numerical targets, human resource development and the system enhancement within 2019." First, at the investigatory level, the plan calls for whole-genome analysis to be performed in a large cohort, the genomic information of Japanese individuals to be compiled at C-CAT, and a system for utilizing this information to be immediately established. By analyzing the enormous amount of whole-genome analysis data compiled in the database using techniques, such as artificial intelligence, it is anticipated that gene alterations that will serve as novel therapeutic targets will be found and that innovative drug discovery and diagnostic methods unique to Japan will be developed.

### Developing genomic medicine

The gene panel tests currently covered by health insurance center on "treatment panels," which focus on detecting gene alterations that are therapeutic targets. However, the gene alterations in tumor cells include changes, such as fusion genes that are characteristic of a particular type of cancer and are closely related to the clinical characteristics, treatment response, and prognosis of the cancer. Consequently, it is anticipated that genomic medicine will also be developed to utilize "diagnostic panels" and "prognosis prediction panels" to determine a treatment strategy based on adjuvant diagnosis and prognosis prediction. In addition, it is anticipated that the indications of comprehensive genome tests, such as panel tests will be expanded, such that "treatment panels" will be indicated not only for patients for whom no standard treatment is available but also for all patients for whom drug therapy.

Furthermore, the scope of application is expected to expand to evaluating treatment response by means, such as early detection of postoperative relapse and detecting minimal residual disease, and to estimating the risk of adverse events by typing pharmacokinetics-related polymorphisms in a patient's normal cells. Moreover, a broader use of comprehensive genomic analysis will result in an accumulation of findings related to gene alterations that underlie cancer predisposition. An understanding and appropriate explanations of the significance of such gene alterations will allow treatment options, periodic testing of healthy variant carriers (surveillance), and prophylactic resection to be considered.

### Off-label uses of drugs

Currently, gene panel tests are used in patients with solid tumors for whom there is no standard treatment or who have completed such treatment (including patients expected to complete treatment). Consequently, the drug therapies recommended by gene panel testing are likely to be often used in a clinical trial or off-label use, and enrollment in a clinical trial is considered wherever possible, based on information provided by sources, such as ClinicalTrials.gov, National Institute of Public Health (NIPH) clinical trial search, and C-CAT reports. If enrollment in a trial or study is difficult, the system in Japan permits administration of an off-label drug in the following circumstances.

1. Treatments for which coadministration with a treatment covered by health insurance is approved

To evaluate a treatment for insurance coverage, off-label use is possible under certain procedures.

- Evaluation treatments (advanced medical care, clinical trials, expanded-access program)
- Patient-proposal healthcare services

(2) Notification No. 51 of the Health Insurance Bureau, Ministry of Health and Welfare, dated September 3, 1980

Off-label use of a drug that has been approved in Japan and has completed the reexamination may be permitted after determining whether it is advisable that the drug is covered by health insurance based on scientific evidence and the drug's pharmacologic action, on a patient-by-patient basis. However, such use is often difficult for novel drugs, such as molecularly targeted drugs.

(3) Other cases

Although safety management measures were established in the Medical Care Act for the use of unapproved drugs, such use has no standing in terms of health insurance.

A mechanism for patient-proposal healthcare services was established in April 2016, in which a treatment begins with a request from a patient for the expeditious use of a drug, etc. that has not been approved in Japan. A patient who desires such a treatment consults with a core clinical research hospital or a special functioning hospital that serves as a liaison for patient-proposal healthcare services and submits a request, along with an implementation plan and other necessary documents to the national government. The implementation plan is reviewed by a patient-proposal healthcare services assessment panel, enabling the patient-proposal healthcare services to be administered.

In November 2018, the patient-proposal healthcare services assessment panel examined how to respond to the fact that a patient for whom gene panel tests identify drug therapies that show promise of efficacy but these therapies cannot be used because administration of a drug covered by health insurance or enrollment in an appropriate clinical study is difficult may apply for a patient-proposal healthcare service; however, in such cases, there are some problems in case-by-case reviews. Consequently, the following mechanism has been established: a research protocol to respond to multiple cancer types and gene alterations is prepared in advance, reviewed by a clinical research review committee and approved by the patient-proposal healthcare service assessment panel, and then shared at core clinical research hospitals. In a July 2019 meeting of the patient-proposal healthcare service assessment panel, some of the molecularly targeted drugs approved in Japan were listed as investigational drugs, and prior deliberations were held regarding a plan to implement patient-proposal healthcare services that can respond to requests for the use of off-label drugs for multiple cancer types or gene alterations. The first such request was approved in September 2019, during the 17th meeting of the patient-proposal healthcare service assessment panel. On October 1, 2019, it became possible to administer "molecular targeted therapy based on gene profiling using multiplex gene panel testing" as a patient-proposal healthcare service. It is anticipated that the drugs that are available upon patients' requests will be expanded in the future.

### Children, adolescents, and young adults

Pediatric cancer affects a small number of patients, and the types of cancer in this group are diverse and rare. Consequently, it includes diseases for which a standard treatment has not been established. On the other hand, the significance of diagnosis, disease classification and prognosis prediction based on the genomic characteristics of tumor cells in administering standard treatment has been established. Consequently, when diagnosing pediatric cancer, gene panel testing is considered during diagnosis and prognosis prediction to determine an adjunct diagnosis or treatment strategy based on genomic findings or to select a therapeutic drug that is likely to show efficacy.

Not only is the incidence of rare cancers, such as germ cell tumors high in adolescents and young adults (mainly 15–40 years old), but standard treatment has often not been established for this group for many types of cancer that are also seen in other age groups, such as leukemia and breast cancer. Gene panel testing is given positive consideration for cancer in adolescents and young adults for which no established standard treatment is considered available based on the patient's age at onset.

Moreover, the proportion of patients with cancer predisposition in their background is high among patients with cancer onset at a young age. Testing is therefore performed at an institution where there is thorough knowledge of hereditary tumors and a genetic counseling system, following an explanation by a specialist counselor. Even when panel testing is performed based on the consent of a legal representative because the patient is young, the patient's "right to know" and "right not to know" in the future is respected as a rule. An opportunity will be ensured to again determine the patient's wishes regarding secondary findings after considering the timing of their disclosure and their significance.

### Center for Cancer Genomics and Advanced Therapeutics (C-CAT)

C-CAT was established as a new center for cancer genomic medicine, an important issue in the 3rd Basic Plan to Promote Cancer Control Programs, which is based on the Cancer Control Act. The center plays a role in compiling and storing information on genomic medicine from across Japan and in managing and improving the quality of genomic medicine. It also provides a mechanism for the appropriate use (secondary use) of that information to create novel treatments (https://www.mhlw.go.jp/file/06-Seisakujouhou-10900000-Kenkoukyoku/0000196975.pdf).

C-CAT compiles and stores patient information (clinical and genomic information) to support genomic medicine for individual cancer patients. Specifically, it supports cancer genomic medicine in Japan by providing C-CAT reports, which determine the significance of gene alterations, to expert panels at designated core hospitals and designated hospitals for cancer genomic medicine, and by understanding the genomic and clinical information of cancer patients in Japan and uses it to formulate cancer control policy. Designated core hospitals, designated hospitals, and cooperative hospitals for cancer genomic medicine provide patient information to C-CAT for the purpose of treatments mentioned above. Even if a patient does not consent to the use of their information for research purposes (secondary use), C-CAT will accept the information if the patient agrees to allow it to be provided to C-CAT, and it will be stored and not deleted even after the patient dies.

#### Registering information with C-CAT

In accordance with a notification from the director of the Medical Economics Division, Health Insurance Bureau, Ministry of Health, Labour and Welfare (HIB/MED Notification No. 0531-1), and with the consent of the patient, designated core hospitals, designated hospitals, and cooperative hospitals for cancer genomic medicine register gene sequence data (FASTQ or BAM) obtained by gene panel testing covered by health insurance, analysis data (VCF or XML), and clinical information with C-CAT (https://www.mhlw.go.jp/content/12400000/000514782.pdf). Consent is obtained using model document of the informed consent form, etc. (including document revised as appropriate) approved by the Liaison Council for Designated core hospitals, etc. for Genomic Medicine and the "Informed Consent Procedures".

Medical information registered with C-CAT before testing includes the patient's sex, age, birthdate, and type of cancer, and this is used as basic information in preparing the C-CAT reports to be used by the expert panel. In addition, information related to baseline characteristics, type of cancer, and drug therapy (e.g., presence or absence of metastasis, pathological diagnosis, ECOG PS, smoking history, family history, genetic testing results, details of drug therapy and the dates it started and ended, and best overall response) is registered before the expert panel is held. The information is presented at the expert panel and used in examining the C-CAT reports and a treatment strategy. Information obtained after the expert panel meeting, regarding the treatment strategy, drug(s) used, adverse events, outcome, date of the last follow-up, date of death, and cause of death, is also registered according to the procedure.

Patient information compiled by the C-CAT from the own institution and other institutions can be used for research purposes after being reviewed by an information utilization review board (tentative name).

## Clinical Questions


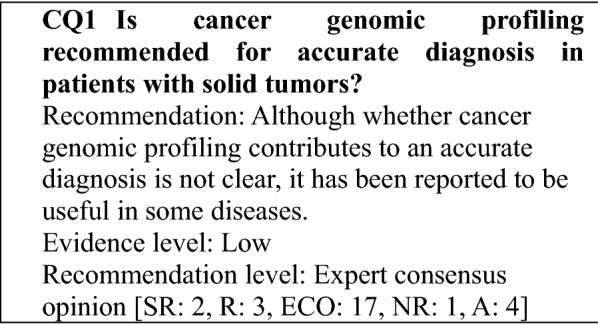


The main purpose of approved cancer genomic profiling tests is to assist in selecting a treatment. They are not intended for the purpose of diagnosis. However, an overseas study of soft tissue sarcomas examined 5,749 patients and reported that the genomic profiling led to a change of diagnosis by detecting characteristic fusion genes in 132 patients (2%) and a more detailed diagnosis of the histological type in 99 patients (2%). In the TOP-GEAR project in Japan, which used the NCC Oncopanel test, *MDM2* amplification was seen in 2 of 187 cases, and the profiling results were found to be useful in diagnosing dedifferentiated liposarcomas [3]. Although the results can be expected to vary depending on the disease and the panel test used, genomic profiling may contribute to an accurate diagnosis in some diseases and will be the topic of future investigation (Table 6).

**Table 6:** Specialists on expert panel and their roles

Panel member	Central role(s)
Cancer drug therapy specialist	C, D
Clinical genetics specialist	E, F
Genetic counseling specialist	E, F
Pathology specialist	A, B
Cancer genomic medicine specialist	B, C, D, E, F
Bioinformatics specialist	A


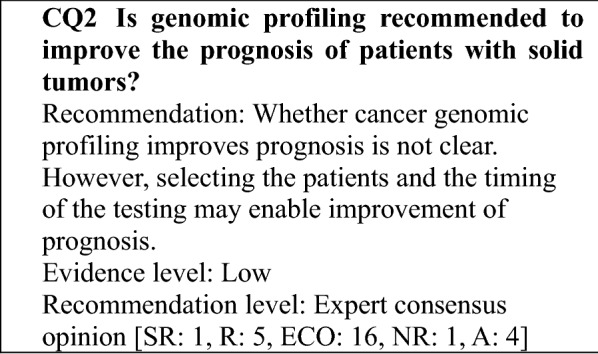


In the SHIVA study, a randomized controlled trial, 195 patients with solid tumors who had completed standard treatment were randomized to a group that received the study treatment (99 patients) or a control group (96 patients). Patients in the study treatment group were administered a molecularly targeted agent to which they were matched based on test results. Those in the control group were administered a drug therapy selected by the investigator (Table 7) [51]. However, no improvement in prognosis was obtained in the study treatment group. On the other hand, in retrospective cohort or case series studies, comparisons with matched control groups or within cohorts suggested improvements in prognosis, although treatment histories and the timing of the testing varied between the studies (Table 8). There have been no reports of randomized controlled studies that have shown an improvement in prognosis with cancer genomic profiling in patients who have not yet completed standard treatment.

**Table 7:** Molecularly targeted agents in the SHIVA study

Targets	Molecular alterations	Molecularly targeted agents
KIT, ABL 1/2, RET	Activating mutation^†^ or amplification*	Imatinib 400 mg qd PO
PI3KCA, AKT1 AKT2, 3, mTOR, RAPTOR, RICTOR PTEN STK11 INPP4B	Activating mutation or amplification Amplification Amplification Homozygous deletion or heterozygous deletion + inactivating mutation or heterozygous deletion + IHC confirmation Homozygous deletion or heterozygous deletion + inactivating mutation Homozygous deletion	Everolimus 10 mg qd PO
BRAF	Activating mutation or amplification Abirterone 1000 mg qd PO	Vemurafenib 960 mg bid PO
PDGFRA/B, FLT3	Activating mutation or amplification	Sorafenib 400 mg bid PO
EGFR	Activating mutation or amplification	Erlotinib 150 mg qd PO
ERBB2/HER2	Activating mutation or amplification	Lapatinib 1000 mg qd PO + Trastuzumab 8 mg/kg IV followed by 6 mg/kg IV q3w
SRC EPHA2, LCK, YES1	Activating mutation or amplification Amplification	Dasatinib 70 mg bid PO
ER, PR	Protein expression > 10%	Tamoxifen 20 mg qd PO (or letrozole 2–5 mg qd PO if contraindicated)
AR	Protein expression > 10%	Abiraterone 1000 mg qd PO

**Table 8:** Investigations of cancer genomic profiling and prognosis

Clinical study	Design	Sample size	Cancer type	Intervention	Results (vs control)
SHIVA [51]	Randomized phase II study	195	Metastatic solid tumors After completion of standard treatment	Molecularly targeted agents	PFS 2.3 vs 2.0 months, HR 0.88, *P* = 0.41
I-PREDICT [52]	Cohort or case series	83	Solid tumors Treated	Molecularly targeted agents, immune checkpoint inhibitors	PFS 6.5 vs 3.1 months (matched > 50% vs ≤ 50%), HR 0.40, *P* = 0.001
LCMC [53]	Cohort or case series	578	Lung cancer	Molecularly targeted agents	OS 3.5 vs 2.4 years, HR 0.69, *P* = 0.006
LCMCII [54]	Cohort or case series	875	Lung cancer	Molecularly targeted agents	OS 2.7 vs 1.5 years
MD Anderson Cancer Center Initiative [55]	Cohort or case series	291	Solid tumors	Molecularly targeted agents	Response 27% vs. 5%; *P* < 0.0001, TTF 5.2 vs 2.2 months, *P* < 0.0001, OS 13.4 vs. 9.0 months, *P* = 0.017
Radovich et al. [56]	Cohort or case series	101	Solid tumors Treatment history of at least 1 regimen	Molecularly targeted agents, chemotherapy, immune checkpoint inhibitors	PFS 86 vs 49 days, HR 0.55, *P* = 0.005
UC San Diego Moores Cancer Center PREDICT [57]]	Cohort or case series	180	Solid tumors	Molecularly targeted agents, endocrine therapy	PFS 4.0 vs 3.0 months, *P* = 0.039, OS 15.7 vs 10.7 months, *P* = 0.04
WINTHER trial [58]	Cohort or case series	107	Solid tumors Subsequent to the standard therapy	Molecularly targeted agents, chemotherapy, immune checkpoint inhibitors	HR 0.482 (ARM A), 0.561 (ARM B)
Princess Margaret IMPACT/COMPACT [59]	Cohort or case series	245	Solid tumors	Molecularly targeted agents, chemotherapy, immune checkpoint inhibitors	Response 19% vs 9%, *P* < 0.026
Von Hoff et al. [60]	Cohort or case series	86	Solid tumors Refractory to 2 or more regimens	Molecularly targeted agents, chemotherapy	PFS ratio ≥ 1.3, *P* = 0.007
Schwaederle et al. [61]	Metaanalysis (phase I studies)	13,203	Solid tumors, hematological neoplasm	Molecularly targeted agents, chemotherapy	Response 30.6% vs 4.9%, *P* < 0.001, PFS 5.7 vs 2.95 months *P* < 0.001
Schwaederle et al. [62]	(phase II studies)	32,149	Solid tumors, hematological neoplasm	Molecularly targeted agents, chemotherapy	Response 31% vs 10.5%, *P* < 0.001, PFS 5.9 vs 2.7 months, *P* < 0.001, OS 13.7 vs 8.9 months, *P* < 0.001

*PFS* progression-free survival, *HR* hazard ratio, *OS* overall survival, *TTF* time to treatment failure

The results can be expected to vary greatly depending on factors, such as subject selection, timing of the testing, the genomic profiling panel used, and access to the subsequent drug therapy. Consequently, it is currently difficult to specify the types of patients who should undergo cancer genomic profiling from the perspective of prognosis improvement, and this is a topic for future investigation.
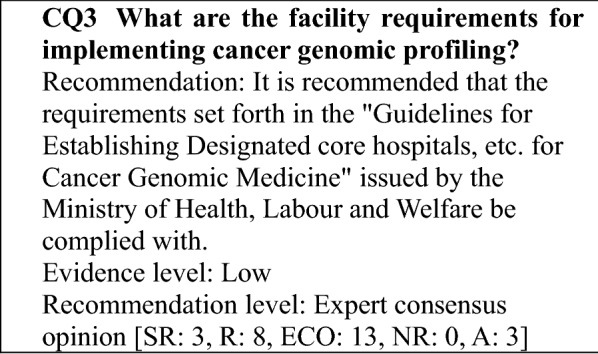


Because a variety of capabilities are required to implement the clinical use of gene panel tests with cancer genomic profiling functions, the Ministry of Health, Labour and Welfare (MHLW) established the "Guidelines for Establishing Designated core hospitals, etc. for Cancer Genomic Medicine" (referred to as the "establishment guidelines" below). In accordance with the requirements specified in the establishment guidelines, the following medical institutions were established (the numbers of facilities shown are as of September 2019).

- Designated core hospitals for cancer genomic medicine (11 facilities: designated by the MHLW; referred to as "designated core hospitals" below)
- Cooperative hospitals for cancer genomic medicine (156 facilities: designated by the MHLW; 34 of these facilities were designated as designated hospitals for cancer genomic medicine in September 2019; referred to as "cooperative hospitals" below)
- Designated hospitals for cancer genomic medicine (34 facilities: designated by the MHLW; referred to as "designated hospitals" below)

The establishment guidelines include the items needed to perform cancer genomic profiling, and adhering to these requirements is recommended.

The period of designation as a designated core hospital or designated hospital extends through March 2022. The subsequent designation period requires further deliberation and has not yet been determined. To add or eliminate medical institutions as cooperative hospitals, the collaborating designated core hospital or designated hospital must submit requests to the MHLW annually.

For the two gene panel tests with cancer genomic profiling functions that were listed in the national health insurance (NHI) reimbursement price list in June 2019, it was required to undergo these tests at medical institutions indicated in the establishment guidelines. Therefore, currently an individual can undergo gene panel tests covered by insurance at designated core hospitals, designated hospitals, and cooperative hospitals (referred to below as "designated core hospitals, etc. for cancer genomic medicine") only.

The establishment guidelines and medical institutions can be seen by clicking on the link to the MHLW website below.

Designated core hospitals, etc. for cancer care.

https://www.mhlw.go.jp/stf/seisakunitsuite/bunya/kenkou_iryou/kenkou/gan/gan_byoin.html

Details regarding the requirements for designated core hospitals, etc. for cancer genomic medicine and the application procedure can be seen by referring to the establishment guidelines. An overview of the capabilities required of such facilities is provided below (Table 9).

In the established system, it is assumed that all of the designated core hospitals, etc. for cancer genomic medicine have the capability to provide care based on gene panel tests (as for an expert panel, which requires specialists, a cooperative hospital requests the designated core hospital or designated hospital, with which the cooperative hospital collaborates, to hold an expert panel), and the patients examined undergo the entire process from testing to receiving an explanation of the results without changing the locations. Designated core hospitals also play a role in advancing cancer genomic medicine. Therefore, they are expected to take the initiative in areas, such as training personnel involved in genomic medicine, developing new genomic tests, and developing drug therapies linked to genes discovered through testing, by collaborating with other medical institutions.

In the 3rd Term Basic Plan to Promote Cancer Control Programs, the Japanese government established the goal of building a system that will enable cancer patients to receive genomic medicine anywhere in the country. Although the establishment of this system will likely continue to progress in stages, the requirements for medical institutions and the system for providing genomic medicine are expected to change flexibly, reflecting the development of new technologies and the associated systemic changes.

Guidelines for Establishing Designated core hospitals, etc. for Cancer Genomic Medicine: https://www.mhlw.go.jp/content/000532262.pdf.
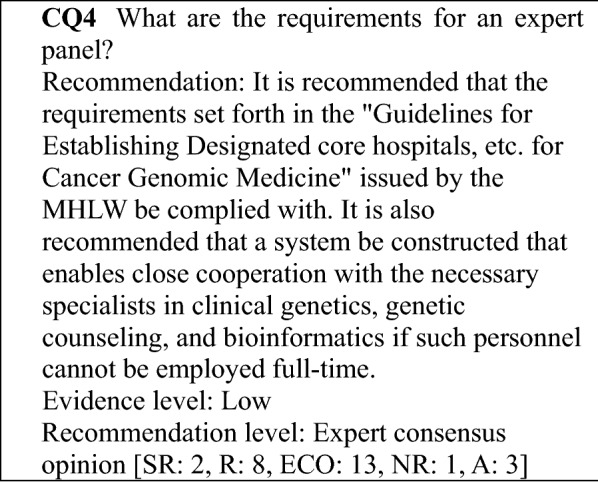


If the number of gene panel tests increases in the future, returning results to patients could be delayed due to conference wait times if the number of facilities that can hold expert panels independently without relying on a designated core hospital for cancer genomic medicine does not increase, preventing the genome data obtained from being utilized for care. The MHLW's "Guidelines for Establishing Designated core hospitals, etc. for Cancer Genomic Medicine" acknowledge that the specialists required to convene expert panels, particularly specialists in clinical genetics, genetic counseling, and bioinformatics, are difficult for many hospitals to employ on a full-time basis because of the limited number of such specialists in Japan. Clinical genetics specialists and genetic counselors play important roles in responding to secondary findings, but since such findings are more central to the care of the patient's relatives than to the care of the cancer patient himself or herself, there is comparatively more leeway in time to disclose such findings. Therefore, if a system that enables the assistance of a clinical genetics specialist or genetic counselor to be obtained when needed has been established, it is not essential that they be employed full-time. The role of bioinformatics specialists is mainly to validate the quality of specimens and data. However, the gene panel tests that have been approved for insurance are performed in laboratories that have precision controls in place. Therefore, there are unlikely to be many cases in which a data quality validation by a bioinformatics specialist is needed.

In summary, for facilities that do not have full-time specialists in clinical genetics, genetic counseling, or bioinformatics, a system that enables the assistance of such specialists to be quickly obtained should be established in order for these facilities to independently convene expert panels.
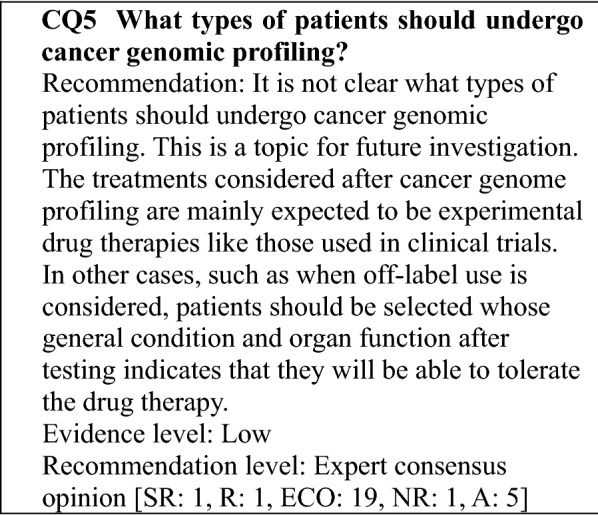


In the SHIVA study, a randomized controlled trial, patients with solid tumors were enrolled after completing standard treatment but showed no improvement in prognosis. On the other hand, improved prognosis as compared with a control group were suggested by the results of retrospective cohort studies. However, although some of these studies limited enrollment to patients with types of cancer, such as lung cancer, many enrolled patients with solid tumors as a whole. Consequently, it is unclear what types of cancer patients see improvements in prognosis as a result of genomic profiling.

Approved cancer genomic profiling tests are covered by the national health insurance for the following patients: "of patients with solid tumors for which there is no standard treatment or patients with solid tumors in whom locally advanced disease or metastasis is seen and who have completed standard treatment (including patients expected to complete the treatment), those who are judged by the attending physician to have a strong likelihood of being suitable for chemotherapy after the test according to the chemotherapy guidelines, etc. of the relevant academic society, based on factors, such as their general condition and organ function." For each patient, the national health insurance point is calculated once.

If there is a companion diagnostic method for the standard treatment for which evidence has been established, the companion diagnosis should be performed first.

The treatments considered after cancer genomic profiling are mainly expected to be experimental drug therapies like those used in clinical trials. In other cases, such as when off-label use is considered, patients should be selected whose general condition and organ function after testing indicates that they will be able to tolerate the drug therapy (Table 9).

**Table 9:** Capabilities designated core hospital s, etc. for cancer genomic medicine

		Designated core hospitals	Designated hospitals	Cooperative hospitals
Capability of providing care based on gene panel testing	Explanation to patients (tests)	Required	Required	Required
	Specimen preparation	Required	Required	Required
	Sequencing	Can be outsourced	Can be outsourced	Can be outsourced
	Holding expert panels	Required	Required	To be requested of a designated core hospital or designated hospital (participation of attending physician required)
	Report preparation	Required	Required	
	Explanation to patients (results)*^1^	Required	Required	Required
	Treatment*^2^	Required	Required	Required
Capability of advancing cancer genomic medicine	Registration with C-CAT*^3^	Required	Required	Required
	Biobank system	Required	Required	Required
	Clinical research and development	Required	Provide cooperation	Provide cooperation
	Personnel development	Required	Provide cooperation	Provide cooperation

*1: Along with explaining the results of gene panel tests, provide and cooperate in genetic counseling as needed

*2: Cooperates with other institutions as needed

*3: Only patients who consent to providing information to C-CAT


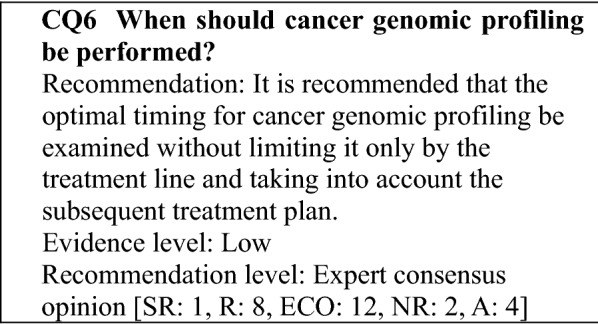


In the SHIVA study, a randomized controlled trial, which examined whether administration of a drug to which the patient was matched based on a comprehensive cancer genomic profiling improved the prognosis of patients who had completed standard treatment, no improvement in prognosis was seen. On the other hand, improvements in prognosis were seen as compared with control groups in retrospective cohort studies, many of which did not include only patients who had completed standard treatment. There have been no controlled studies that have examined the timing of cancer genomic profiling with which improvements in prognosis can be expected, and this is a topic for future investigation. However, although it should be noted that there were differences with respect to the subjects and endpoints in these studies, a randomized controlled trial limited to patients who had completed standard treatment did not indicate the efficacy of cancer genomic profiling, while a study not limited to patients who had completed standard treatment suggested efficacy. Thus, there is little scientific basis for restricting cancer genomic profiling to patients have completed standard treatment.

In patients with solid tumors for which a standard treatment has not been established, such as rare cancers and cancers of unknown primary, it is recommended that testing be performed before the start of treatment to assist in selecting a treatment.

It is recommended that the optimal timing for cancer genomic profiling be examined without limiting it only to the treatment line but rather taking into account the plan for subsequent treatment. This is based on the fact that some clinical studies of novel drugs examine the initial treatment, even in types of cancer for which there are multiple standard treatments; the fact that the test results may affect the determination of the treatment plan; and the fact that factors such as the patient's general condition and organ function may worsen while waiting for the standard treatment to be completed, eliminating the opportunity for treatment.
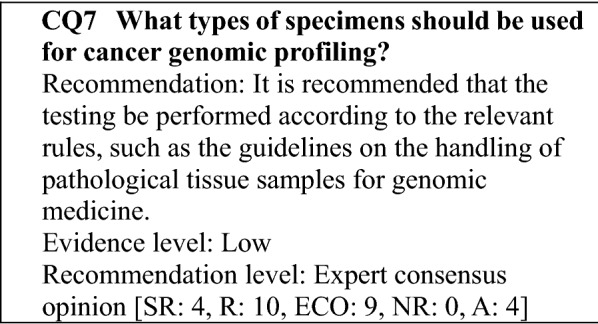


Formalin-fixed paraffin-embedded (FFPE) samples used for routine pathological diagnosis are used in the gene panel test for cancer genome profiling. However, to obtain high-quality DNA, attention should be paid to tissue collection, fixation, storage, and the percentage tumor cell content and tumor cell content in specimens provided for testing.

The percentage tumor cell content is the proportion of tumor cell nuclei among nucleated cells in the tissue to be analyzed. Since this is sometimes confused with tumor cell occupancy (proportion in terms of area), caution is needed in this regard.
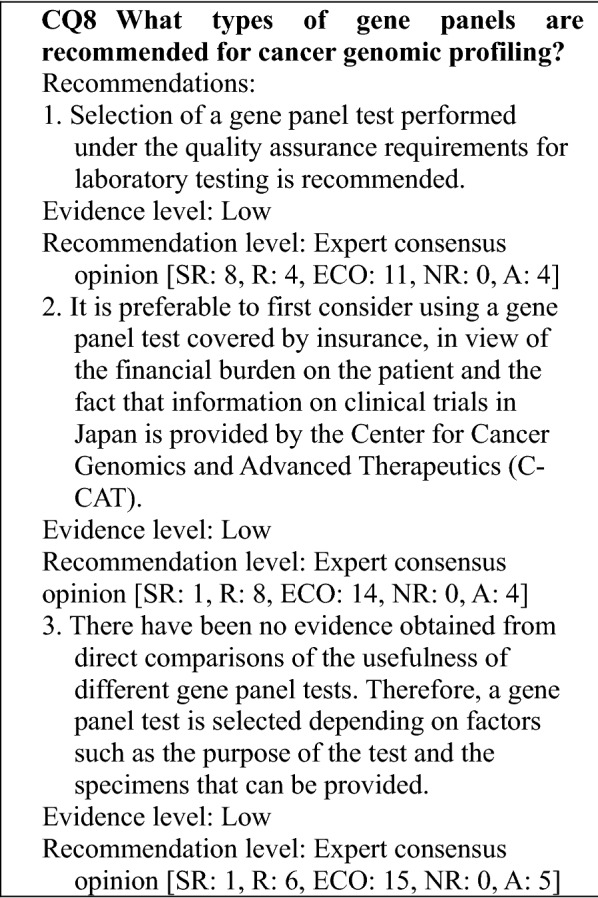


Explanation:

1. Most genetic analysis performed using next-generation sequencers has been implemented for research. However, quality assurance of laboratory testing is required to perform gene panel tests in the course of clinical care and to use the results in the care of patients, and in October 2018, the Japanese Promotion Council for Laboratory Testing issued the "Basic Concepts for Ensuring the Quality and Precision of Cancer Gene Panel Tests" (version 2.0, May 31, 2019) [19].

In the United States, analyses must be performed in clinical laboratories certified according to the provisions of the Clinical Laboratory Improvement Amendments (CLIA), which are laws that establish quality standards for clinical laboratories. In Japan, ISO 15,189 and the Laboratory Accreditation Program of the College of American Pathologists (CAP) are used as the clinical laboratory evaluation standards. In a Q & A document issued by the Medical Economics Division, Health Insurance Bureau, MHLW on June 4, 2019, the following question and answer are presented: "Regarding the FoundationOne^®^ CDx Cancer Profile and OncoGuide™ NCC Oncopanel System, which were covered by insurance as of June 1, 2019, the amended Points to Consider Notification dated May 31, 2019 states the following: 'To perform the testing, the measures required to ensure the quality and precision of tests performed using sequencer systems must be established, and the testing must be performed at an insurance medical institution that has been accredited by an appropriate third party involved in testing using sequencer systems. If the testing is subcontracted to a clinical laboratory, the laboratory should also have a similar third-party accreditation.' In this instruction, what does 'an appropriate third-party accreditation' refer to?" To this question, the answer provided states: "Currently, the CAP's Laboratory Accreditation Program corresponds to such a third-party accreditation. If an appropriate new accreditation system is identified, it will be publicly announced."

2. In Japan, a large number of gene panel tests are implemented, including those not covered by insurance. With such uncovered tests, the cost to the patient may be 500,000 yen or more, which is not insignificant. In 2018, C-CAT was founded for the purpose of collecting, managing, and utilizing genome data from testing performed under insurance-covered medical care. For patients who consent to registration in C-CAT, Japanese clinical trial information corresponding to obtained gene alterations is extracted from the "integrated knowledge base for genomic medicine" created by C-CAT and provided to expert panels as C-CAT reports. Because this is useful information for the patient, it is preferable to first consider gene panel tests that are covered by insurance.
3. There has been no evidence obtained from direct comparisons of the usefulness of gene panel tests that are covered by insurance or used in the advanced medical care. On the other hand, the amount of specimen required, the eligibility requirements for specimens, and the capabilities of the test differ in part by the gene panel test (see Table 2). Therefore, a gene panel test should be selected according to the status of the specimens that can be provided and the purpose of the test, based on a thorough understanding of the characteristics of each test.

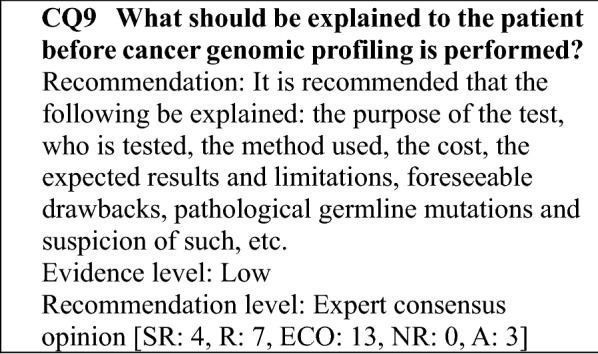


There have been no controlled studies that have examined the information provided to patients about cancer genomic profiling, how well the patient and their family members understand the information, and their reaction to the information.

The information that should be provided to patients is elaborated on below.

1. The purpose of the cancer genomic profiling

Explain why the test will be performed. The cancer genomic profiling tests that have been approved are those used to explore appropriate drug therapies and therapeutic methods by comprehensively examining large number of cancer-related genes.

2. Patients who undergo cancer genomic profiling

Explain who will undergo cancer genomic profiling. The indication of currently approved cancer genomic profiling tests is "of patients with solid tumors for which there is no standard treatment or patients with solid tumors in whom locally advanced disease or metastasis is seen and who have completed standard treatment (including patients expected to complete the treatment), those who are judged to have a strong likelihood of being suitable for chemotherapy after the test according to the chemotherapy guidelines of the relevant academic society, based on factors, such as their general condition and organ function."

3. Method used to perform cancer genomic profiling

The information provided to the patient should also include the following: the types of specimens used (e.g., tissue, blood), the method of collection (whether new specimens will be collected or existing specimens used, etc.), the type of gene panel used (if testing can be performed, what can and cannot be detected, etc.), the risks and cost associated with the test, the possibility that the test may ultimately be unsuccessful, and the fact that designated core hospitals, designated hospitals, and cooperative hospitals for cancer genomic medicine will share information to enable a detailed examination of the analysis results.

4. Results obtained from cancer genomic profiling

For each test objective, explain what types of results can be expected. If the genomic profiling would be performed to explore appropriate drug therapies and therapeutic methods, explain how likely it is that a drug therapy will be identified. At the same time, also explain beforehand that there is strong possibility that a drug therapy cannot be identified and that the patient should therefore consider whether they wish to undergo the test. It is also necessary to explain beforehand that even if an appropriate drug is identified as a result of the test, it may not be a treatment option in cases, such as the following.

- The drug has not received marketing approval in Japan.
- The drug has not received an indication for the type of cancer the patient has.
- The drug has been used only in clinical studies, and the patient does not meet the eligibility criteria..

5 Pathological germline mutations that may be identified by performing cancer genomic profiling and the suspicion of such

Depending on the test performed, pathological germline mutations and the suspicion of such should be examined. The following should be explained prior to the test: what a pathological germline mutation is, what can be expected, whether the patient wishes for a pathological germline mutation or the suspicion of such to be disclosed, matters concerning the individual to whom such information would be disclosed, and genetic counseling and additional genetic tests.
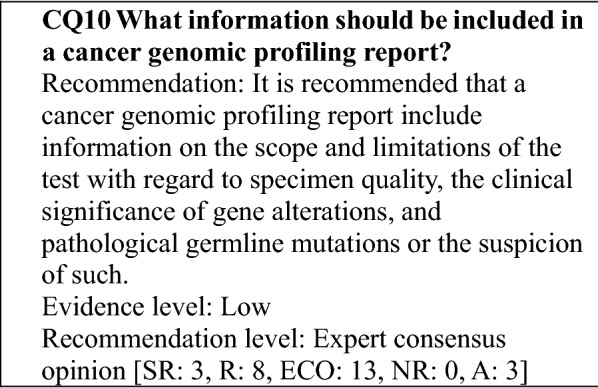


There has been little reported on the content of cancer genomic profiling reports.

As the consensus of the Clinical Genome Resource (ClinGen) working group in the United States, the minimum variant level data (MVLD) were proposed as the minimum information to be provided when evaluating the clinical significance of genetic alterations. The MVLD include the following: the version of the reference genome, gene name (HUGO gene nomenclature), gene position (HGVS nomenclature), somatic cell/germline distinction and whether determined, alterations of genes and amino acids (HGVS nomenclature), type of anomaly (e.g., SNV, missense), information on clinical significance (e.g., type of markers for response prediction/prognosis prediction/diagnosis, evidence level), and the PMIDs of articles serving as evidence. Although the MVLD constitute the standard for registering clinical evidence related to genetic alterations in the knowledge base, it can also be referenced for cancer genomic profiling reports [63].

All tests have limitations, and it is difficult to expect cancer genomic profiling to be perfectly accurate or comprehensive. Even if a test is considered the best at the time it is implemented, scientific and technical advances may enable more extensive anomalies to be detected and more appropriate evaluations to be performed in the future. Because the test results can be expected to be reviewed again later, it is best to indicate the scope and limitations of the test in the report so that this information is available for review.

In preparing this guidance, the following items were listed as desirable to indicate in reports by testing facilities and reports by expert panels.

**Reports by testing facilities (including C-CAT reports)**

o Genes targeted, scope of sequencing,^1)^ types of anomalies^2)^
o Whether the report should include the results on germline anomalies (if it should includes partially, indicate to that effect)
o Disease name, organ from which specimen collected, date specimen collected, tumor cell percentage^3)^
o Test start date, quality of specimens, such as DNAs
o Details on gene alteration detected,^4)^ specimen in which detected^5)^
o Biological significance of gene alteration detected^6)^
o Specific candidate drug(s) for gene alteration and evidence level
o Indication(s) of candidate drug(s) and availability rank based on clinical trial information^7)^
o Presence/absence and significance of pathological germline mutations or suspicion of such
o Types of databases used to determine significance^8)^ and dates accessed
o Points to consider that the evaluations of detected gene alterations and their clinical significance, etc. depend on the test method used, programming, and the databases referenced and may change in the future

(1) The entire coding region of the gene or a specific region; (2) whether any of fusion, amplification, TMB, MSI, etc., is included; for amplification, its definition; (3) if part of a specimen has been selectively resected (dissection), indicate to that effect; (4) including type of anomaly and variant allele frequency; (5) whether derived from somatic cell or germline; (6) pathological mutations, etc.; (7) ease of accessing treatments; and (8) genetic polymorphism database, knowledge base that compiles evidence for candidate drugs, and other.

**Reports by expert panels**

- Whether there is a recommended treatment and a description of any such treatment
- Treatment options other than the recommended treatment
- Whether there are germline mutations for which an explanation to the patient is recommended and descriptions of any such anomalies
- Revisions and additions to reports prepared by testing facilities, etc.
- Sources used as evidence
- That although the review of the expert panel is based on the patient's treatment history, treatments reviewed are other than the standard treatment, and that it is the attending physician's responsibility to judge the implementation of standard treatment
- That the conclusions of the expert panels are based on the scientific knowledge and clinical study information currently available and may change as new information is obtained in the future.

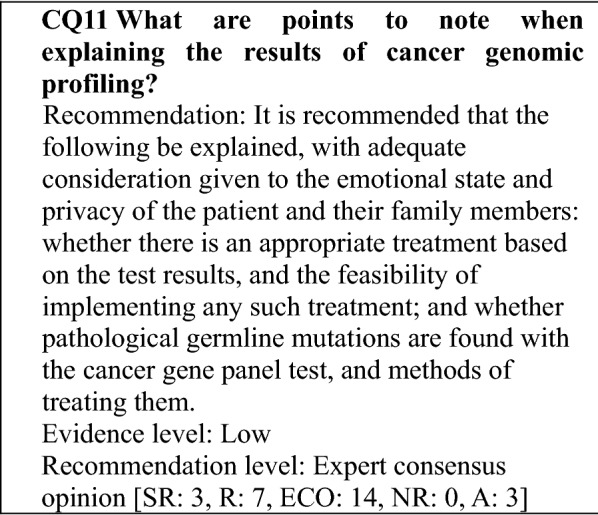


There have been no controlled studies that have examined the important points to note when explaining cancer genomic profiling results and the reactions of patients and their family members to the explanation.

When explaining the results, set aside adequate time for the explanation and arrange an environment that takes privacy into account.

In disclosing the results, explain their significance and the treatments and approaches recommended based on the results of the expert panel's review of the test results obtained, and in line with the information explained before the test (see CQ9). Explain that although an appropriate drug is often not found, even if one is found, it often may not be a treatment option for the reasons raised under CQ9.

Before explaining a pathological germline mutation or the suspicion of such, first confirm whether the patient wishes to have the information disclosed and whether they have family members with whom they wish to share the test results. In addition, it is recommended that the patient be told whether there were secondary findings and, as necessary, that information be provided on genetic counseling or the need for supplemental genetic testing.

If the patient is a child, adolescent, or young adult (see "*3: Confirmation of willingness in child, adolescent, or young adult patients" in "Genetic counseling"), effort will be made to obtain the informed assent of the subject himself or herself, in addition to the informed consent of the patient's parent or legal guardian.

Because the test results may not necessarily lead to an effective response, such as a treatment, it is recommended that the emotional state of the patient also be considered.
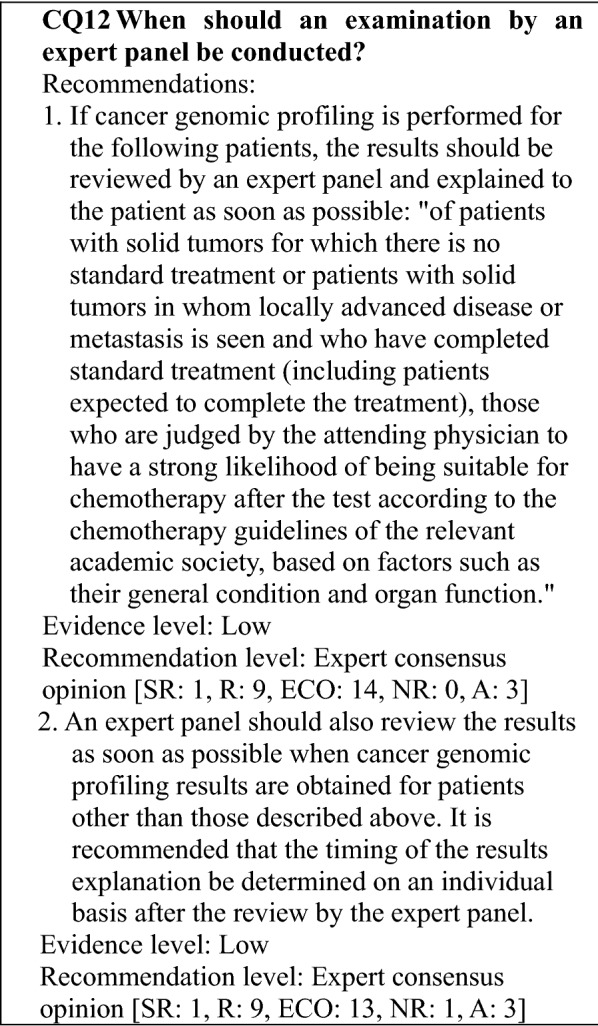

1. It is expected that cancer genomic profiling will be performed to assist in selecting treatments for patients with solid tumors for which there is no standard treatment or for which the standard treatment has been completed. In order not to miss the opportunity for treatment, and to enable the results to be quickly provided to the patient, the expert panel should review the test results and they should be explained to the patient as soon as possible.
2. Currently (as of September 2019), the following rule is specified: "The national health insurance point can also be calculated when the results for a comprehensive genome profile obtained in conjunction with an assessment of a specific gene mutation, which was performed to select an anticancer drug treatment, are provided to the patient after being reviewed by an expert panel following the completion of standard treatment and a written explanation of the treatment strategy, etc., is provided to the patient."

If, for any reason, the results of cancer genomic profiling have already been received for a patient, delaying the expert panel review until after the completion of standard treatment and waiting to provide the results to the patient are not permitted from scientific and ethical perspectives, because such actions could limit the patient's treatment options, delay a response to information that ought to be addressed, such as secondary findings, or result in delayed or insufficient information provision. It is preferable for the expert panel to review the test results as soon as possible. With regard to when to provide the results to the patient, it is preferable to respond on an individual basis following the review by the expert panel and after deciding what results to provide quickly and what results require further appropriate discussion.
